# A novel biclustering algorithm for mining m^6^A co-methylation patterns based on beta-binomial distribution and data screening strategy

**DOI:** 10.1371/journal.pcbi.1014430

**Published:** 2026-06-23

**Authors:** Zhaoyang Liu, Yuteng Xiao, Dao Xiang, Hao Shi, Kaijian Xia

**Affiliations:** 1 School of Information Engineering (School of Big Data), Xuzhou University of Technology, Xuzhou, China; 2 School of Computer Science and Technology, Qilu University of Technology (Shandong Academy of Sciences), Jinan, China; 3 Department of Hematology, Xuzhou Central Hospital, Xuzhou, China; 4 Center of Intelligent Medical Technology Research, Changshu Affiliated Hospital of Soochow University, Suzhou, Jiangsu, China; 5 Beijing Institute of Technology Research Institute of Frontier Technologies, Jinan, Shandong, China; Indonesia International Institute for Life Sciences, INDONESIA

## Abstract

Studies have shown that m^6^A plays a key role in different life processes such as RNA metabolism, physiology and pathology. However, due to the complexity of life processes, its specific regulatory details are still not revealed. The computational approach based on co-methylation pattern mining of m^6^A sequencing data can assist in revealing its mechanism and save time and economic cost, however, the current algorithms suffer from the problems of insufficient robustness to low signal-to-noise data and unreliable performance. Based on this, this paper proposes an enhanced beta-binomial distribution biclustering algorithm (EBBM) based on data screening strategy. This algorithm is based on the framework of Bayesian, adopts Gibbs sampling method for parameter inference, and introduces the data screening strategy in the process of parameter inference, which effectively removes the problem that the low signal-to-noise data in the original sequencing data of m^6^A affects the reliability of the clustering results. The simulation experiment results show that this algorithm can effectively deal with the interference of low signal-to-noise data and accurately mine the co-methylation patterns pre-planted in the data, which is significantly better than the current mainstream biclustering algorithm. In real human m^6^A sequencing data with 32 samples, this algorithm mined two effective co-methylation patterns, which were enriched to different biological processes, such as negative regulation of phosphorylation and peptidyl lysine methylation, etc. The scoring results of GEO_Score indicate that the results of this algorithm are more biologically meaningful than the clustering results of current mainstream m^6^A co-methylation pattern mining algorithms.

## Author summary

Methylation of RNA molecules—specifically a modification known as m⁶A—plays a crucial role in how our cells function, influencing everything from normal development to diseases like cancer. However, studying these modifications is challenging because the sequencing technology used to detect them produces noisy data, making it difficult to distinguish genuine biological signals from technical errors. We developed a new computational approach called EBBM that tackles this problem head-on. Our method works like a smart filter, simultaneously analyzing two complementary datasets generated by sequencing experiments. By incorporating a statistical model that accounts for the unique characteristics of this data, EBBM can identify patterns of co-methylation—groups of RNA sites that are modified together under specific conditions—while effectively discarding sequencing noise. When we tested EBBM on both simulated and real human data, it significantly outperformed existing methods. It successfully uncovered biologically meaningful co-methylation patterns that were linked to processes like cell differentiation and gene regulation. Our work provides researchers with a more reliable tool for studying RNA modifications, potentially accelerating discoveries about how these modifications contribute to health and disease, and opening new avenues for therapeutic development.

## 1. Introduction

N⁶-methyladenosine (m⁶A) methylation is the most abundant epitranscriptomic modification in eukaryotic RNA, which dynamically regulates gene expression through the “write-erase-recognize” mechanism, and plays a key role in different life processes, including RNA metabolism, physiology and pathology. For instance, m^6^A is involved in RNA splicing, export, stability, translation and localization [1,2]. It also regulates circadian rhythms, adipogenesis, spermatogenesis, embryonic stem cell self-renewal and differentiation, neurodevelopment, and neuronal diseases [3–5]. Aberrant m^6^A regulation has also been associated with various cancers including acute myelogenous leukemia [6,7], breast cancer [8–10], pancreatic cancer [11], gastric cancer [12,13], prostate cancer [14], renal cancer [15], mesothelioma [16], sarcoma glioblastoma [17,18], mesothelioma [19,20], lung cancer [21], and hepatocellular carcinoma [22–31]. Depletion of METTL3 is known to lead to apoptosis and reduce cancer cell invasiveness [32,33], while hypoxia-activated ALKBH5 leads to cancer stem cell enrichment [34]. As FTO is a key regulatory gene for energy metabolism and obesity, studies of single nucleotide polymorphisms in FTO have been shown an association with body mass index as well as with the development of obesity and diabetes mellitus in humans [35–39]. In addition, it has also been suggested that FTO affects preadipocyte differentiation [40–42].

However, due to the complexity of life processes, their specific regulatory details remain unrevealed. Currently, with the development of Methylated RNA Immunoprecipitation with Next Generation Sequencing (MeRIP-Seq) [43] and High-Throughput Sequencing [44], more and more methods of wet experiments are being used to detect relevant m^6^A methylation modification in tissues or tumors, and used to annotate the modification changes of m^6^A under different conditions to gradually reveal its functional mechanism. MeRIP-Seq technology is mainly based on the principle of chromosomal immunoprecipitation (IP), utilising specific antibodies to enrich RNA fragments with m^6^A modification and combining this with high-throughput sequencing to locate the methylation sites. Total RNA is first extracted from excised tissue or tumor cells and divided equally into two parts, one part is then randomly fragmented randomly into small fragments of 100–200 nt using chemically or enzymatically methods. This fragmented RNA is then incubated with the fragmented RNA using an anti-m^6^A antibody that specifically binds to methylation sites, and the methylated RNA fragments are enriched by capturing the antibody-RNA complexes by magnetic beads (e.g., Protein A/G) that elute unbound RNA. Finally, the immunoprecipitated RNA (IP) sample and another portion of unenriched control RNA (input) sample are reverse transcribed, library constructed and sequenced separately. Therefore, MeRIP-Seq sequencing technology ultimately describes the m^6^A modification by IP and input samples, which is significantly different from the traditional RNA sequencing technology. On the basis of quality control and data comparison of the sequencing data, the m^6^A modification peaks have to be identified by comparing the signal difference between the IP and input samples, and then analyzing the distribution of the methylation sites in conjunction with gene annotation. Therefore, traditional wet-lab methods can be effective for methylation profiling, yet these methods tend to have high economic and time costs [45]. In recent years, a large amount of sequencing data has been accumulated from biological wet experiments under different conditions. The methods of computation developed based on these data combined with artificial intelligence technology play an increasingly important role in revealing its regulatory laws, and the application of computational methods not only saves economic costs, but also greatly accelerates the study of the functional mechanism of m^6^A [46]. In the exploration of computational methods for m^6^A regulatory law revealing studies, many important works have been proposed based on different levels. Firstly, a series of databases were constructed based on existing biological wet experiments, which paved the necessary research foundation for the study of computational methods. Such as RMBase [47,48], MeT-DBV2.0 [49], m6Avar [50],m6A2Target [51] and m6A-Driver [52]. The above databases cover multi-dimensional research needs from basic mechanisms to clinical translation, and from common transcripts to non-coding RNAs, which provide powerful data support for precisely analyzing the biological functions of m^6^A. On the basis of the above data, a series of tools and prediction algorithms have been proposed, and the following are some representative tools. sRAMP [53] proposed by Cui et al. based on sequence features (K-mer frequency, RNA structure) can perform accurate prediction of m^6^A sites on human and mouse mRNAs. Meng et al. proposed a Whistle [54] model based on the random forests and integration of MeRIP-seq, RNA-seq and CLIP-seq data predicted tissue-specific m^6^A sites. Cui et al. recently proposed another combined framework deepSRAMP [55] based on the transformer architecture and recurrent neural networks, whose prediction accuracy is greatly superior to other state-of-the-art prediction tools such as WHISTLE.

The series of site prediction algorithms constructed above try to decompose the spectrum of m^6^A modification under various conditions at the computational level, and then analyze the correlation between the genes where the site are located and diseases, so as to construct the interaction network between m^6^A and diseases. However, the process of dynamic modification of m^6^A methylation, as well as the occurrence and development of different life processes such as diseases, is a complex and comprehensively regulated by various factors, and it is necessary to explore its potential laws from multiple dimensions, such as time and space, in order to reveal the mechanism of m^6^A modification that drives the progression of diseases through the spatio-temporal specific regulation of gene expression network, and to provide a theoretical basis for precision intervention.

In recent years, the study of m^6^A co-methylation pattern mining has played an increasingly important role in the study of its temporal and spatial synergistic and specific regulatory functions. Co-methylation pattern refers to the phenomenon that multiple m^6^A sites are densely distributed on mRNA molecules at the same time point, which may synergistically regulate RNA stability and translational efficiency, or some non-coding RNAs (e.g., IncRNA MALAT1, circRNA CDR1as) share methylation regulatory elements with mRNAs, which may affect the activity of methylase-reading proteins through the mechanism of “competitive binding”, forming a trans-RNA regulatory network [56–60]. The occurrence of these co-methylation patterns can be over-expressed or under-expressed at the m^6^A modification level at the same time. Therefore, these co-methylation patterns can be mathematically modeled as clusters, and the sites contained in the clusters show the same or similar methylation modification trends under certain conditions, while the clusters differ from each other greatly.

In the study of mining m^6^A co-methylation patterns, Liu et al. first proposed the concept of co-methylation patterns based on MeRIP-Seq data [56]. Based on the work of Liu et al. Cui et al. proposed a hierarchical graph model for clustering peaks in MeRIP-Seq data based on the distributional characteristics of m^6^A high-pass sequencing data [61]. Subsequently Chen et al. proposed another hierarchical clustering algorithm for m^6^A-Seq co-methylation patterns based on threshold weighting in response to the problem of introducing noise in calculating methylation levels [62]. The algorithm modeling reasonably assumes that the modification intensity of m^6^A sites is expressed by dividing the immunoprecipitated samples (IP_s_) characterizing m^6^A modifications by the sum of IP_s_ and input control samples (input_s_), which is also known as the methylation level, i.e., the methylation level of the site s is expressed as IP_s_/(IP_s_+input_s_). To address the problem that when a site has 100 IP reads and 1 input reads under the corresponding conditions, and the methylation level with 1 IP reads and 0 corresponding input reads are both 1, they are all treated as hypermethylated in the clustering process for the calculation, the method of designing the weights is used to integrate the effect of the gene expression into the process of calculation. In this framework, unreliable measurements with a small number of reads counts will be given a smaller weight, while reliable measurements with a larger number of reads counts will be given a larger weight, thus solving the problem of such noise affecting the reliability of the results. Zhang et al. have also recently proposed a nonparametric beta-binomial mixing model, DPBBM, based on the distributional characteristics of the m^6^A data [63]. This model can automatically determine the number of clusters. Liu et al. constructed a single clustering algorithm MBMM based on the beta mixture model using the framework of the EM algorithm and the parameter inference method of moment estimation [64], and mined seven effective co-methylation patterns on human m^6^A modification data. The above algorithms discovered co-methylation patterns hidden in MeRIP-Seq data at a certain level. However, all of the above clustering algorithms are traditional single clustering methods, and they define co-methylation patterns in a relatively simple way, assuming that each co-methylation pattern must contain all sample conditions, and that each site must and only be assigned to one co-methylation pattern. However the above assumptions are biologically unrealistic [65]. Due to enzyme-specific regulation and condition-specificity effect, it is possible that some sites are only locally co-methylated under some conditions. Not all sites have to be assigned to the co-methylation pattern, and it is also possible that there is overlap between co-methylation patterns in terms of sites or conditions. Therefore, traditional single clustering algorithms cannot tap these local co-methylation patterns, and the biclustering algorithms emerged in recent years can solve this problem.

In the study of m^6^A local co-methylation pattern mining by biclustering, based on the m^6^A methylation level data, Zhang et al. proposed an ISA biclustering algorithm based on RNA expression level weighting, REW-ISA [60] and a weighted Plaid biclustering algorithm based on Lagrange multiplier method, FBCwPlaid [66], respectively, on the basis of ISA algorithm and the plaid model, the first to achieve m^6^A local co-methylation pattern mining and discover the potential functional patterns of m^6^A. On the basis of this work, Liu et al. modeled from the dimension of the distributional features of the MeRIP-Seq data, and proposed a beta-mixture distribution-based biclustering algorithm, BDBB [57], which mined two effective local co-methylation patterns on human m^6^A modification data.

The above single clustering and biclustering methods have mined effective co-methylation patterns from different perspectives. However, in terms of data processing, they all transform the original MeRIP-Seq sequencing data, i.e., the matrix of reads in the Ip and input samples is computationally converted into a single methylation level data matrix, that can be handled by algorithm, and such a transformation inevitably introduces noise. Although each algorithm adopts relevant noise reduction methods to a certain extent, it will inevitably affect the accuracy of the results. To address this problem, Liu et al. proposed a biclustering algorithm BBM [58] based on the beta-binomial distribution, which can operate on both matrices of IP and input samples at the same time and mine the co-methylation patterns. BBM defines the biclustering model under the Bayesian framework, adopts Gibbs sampling methods for parameter inference, it realizes that the biclustering operation can be carried out directly on the data of IP samples and input sample reads, by reasonably assuming that the number of IP samples reads follows a beta-binomial distribution, which avoids the problem of noise introduced by biclustering algorithms such as BDBB due to the need of methylation level calculation. BBM overcomes to some extent the problem of noise introduced by traditional methods, which is caused by the computation of methylation levels. However, due to the inherent errors in sequencing technology, measurement data with a low IP/input ratio are often considered noise, the sites may not be methylated in such cases, and it is inappropriate to describe the methylation modification of the sites using this measurement data. BBM does not consider the elimination of such noise effects. To obtain reliable measurement data, the IP/Input ratio is typically used to distinguish methylation signals from background noise, a methodological basis found in the m⁶A MeRIP-seq field. This method assumes that after immunoprecipitation, the RNA fragment containing the true methylation site should be significantly enriched in the IP sample; therefore, the number of reads in the IP sample should be higher than in the Input sample. By calculating the ratio (or derived indicators such as the signal-to-noise (S/N) ratio), the signal can be distinguished from background noise. In the pioneering paper, m^6^A peaks were identified by setting an enrichment threshold of more than fourfold [67]. In [68], while optimizing the refined RIP-seq protocol, Zeng et al. explicitly used the S/N ratio (S/N = (positive region IP ÷ Input) ÷ (negative region IP ÷ Input)) to assess data quality, achieving a signal-to-noise ratio of approximately 100-fold under optimized conditions. The MeTPeak algorithm includes a built-in condition for the IP/Input ratio: ‘mean ratio = IP/(IP+Input) > 0.5’, which is equivalent to IP/Input > 1 [69]. The MeTDiff R package uses ‘FOLD_ENRICHMENT = 1’ by default, requiring that the IP/Input enrichment fold be ≥ 1 [70]. In summary, “IP/Input > 1” is the minimum consensus standard for identifying methylation sites in this field. Typically, different laboratories use different parameters, with 2X and 1.5X enrichment being the most common. However, there is currently no complete standardization. Typically, “1.5X” can be regarded as an empirical conservative value based on this standard. It serves as an industry convention or the default parameter for specific analytical workflows. Furthermore, the sensitivity analysis of threshold data selection in this study also shows that 1.5X is a relatively ideal choice, as it can effectively remove noise while retaining most of the biologically meaningful patterns.

To address above problem, this study selected a threshold of 1.5 times to remove noise and constructs an Enhancing Beta-binomial-distribution biclustering algorithm (EBBM) based on data screening strategy to achieve the mining of local co-methylation patterns of m^6^A. The EBBM can guide the data flow by introducing an algebraic approach into the construction of statistical models, thus achieving the purpose of effective noise removal. Simulation studies show that EBBM can effectively identify the sequencing noise hidden in MeRIP-Seq simulation data. Recovery and relative scoring results show that EBBM is significantly better than the current mainstream co-methylation pattern mining algorithms. On the real IP and input samples reads counts data, EBBM found two effective m^6^A local co-methylation patterns, and the data heatmap shows that their average methylation levels are both greater than 0.6, indicating that EBBM effectively removes the influence of MeRIP-Seq sequencing noise. The GOE_Score scoring results show that EBBM’s scores are significantly higher than those of the current mainstream algorithms, indicating that the patterns found by EBBM contain less noise, and its results are more biologically meaningful than the current mainstream clustering results.

This study makes three significant contributions:

1) Methodological innovation: We develop a novel algebraic-statistical hybrid model under a Bayesian framework, implementing an efficient Gibbs sampling algorithm that establishes a new computational paradigm for MeRIP-Seq data analysis.
2) Technical advancement: Our approach demonstrates superior noise-reduction capabilities, effectively addressing the critical challenge of signal-to-noise ratio improvement in epitranscriptomic profiling.
3) Biological insight: Through rigorous validation, we identify two co-methylation patterns that show higher biological consistency than predictions from existing methods.

## 2. Results

### 2.1. Simulation data experimental analysis

**Fig 1:**
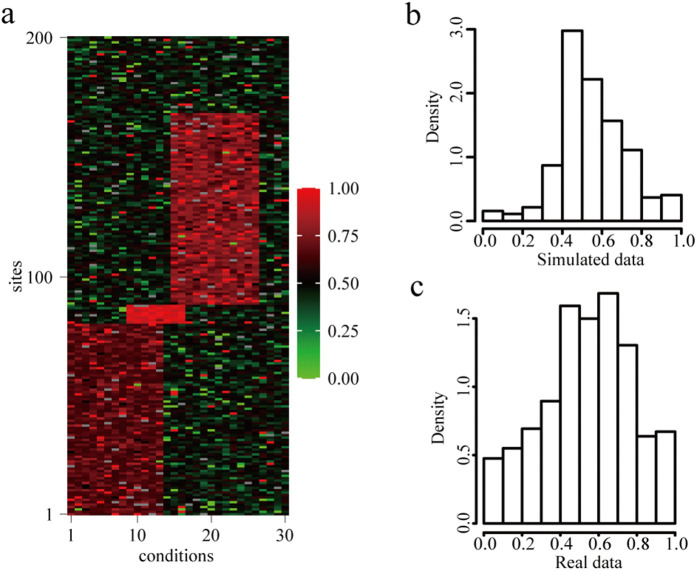
Comparison of Statistical characteristics between simulated data and real data. a Heatmap of simulated methylation level data. b Histogram of simulated methylation level data. c Histogram of real methylation level data.

In the experiment, we first simulated and generated experimental data for 200 sites under 30 experimental conditions, including IP sample reads count data and input sample reads count data. Three biclusters were embedded in the data. In order to better simulate the real-world scenario of m^6^A methylation modification, the parameters of the binomial distribution followed by the three biclusters, as well as the size of the generated data, were repeatedly adjusted to make the characteristics of the simulated data distribution as similar as possible to those of the actual MeRIP-Seq data. The final binomial distribution parameters for the three biclusters were determined to be 0.65, 0.94 and 0.75 respectively. The binomial distribution parameter followed by the background was 0.5. The three biclusters contained 80, 8, and 80 sites, respectively, and in terms of sample conditions, they contained 13, 8, and 12 sample conditions, respectively. The first and second biclusters had five conditions overlap and the second and third biclusters had three conditions overlap, as shown in Fig 1a. The overall distribution characteristics of the simulated data are shown in Fig 1b, and those of the real data are shown in Fig 1c.

Subsequently, the EBBM model was applied to the aforementioned simulated dataset, with the initial iteration count set to 1000, the burn-in count set to 500, the predefined number of biclusters set to 10, and the intra-chain variance threshold set to 0.1. Finally, the EBBM model output three biclusters. When examining the sites and conditions contained in the three biclusters, it was found that the predefined three biclusters were accurately reproduced, and the overlaps in conditions between them were also accurately reproduced. When checking the values of the binomial distribution parameter ρ output by them, it was found that they also approach the ground-truth, specifically 0.6469045, 0.9419758, and 0.7525028.

**Fig 2:**
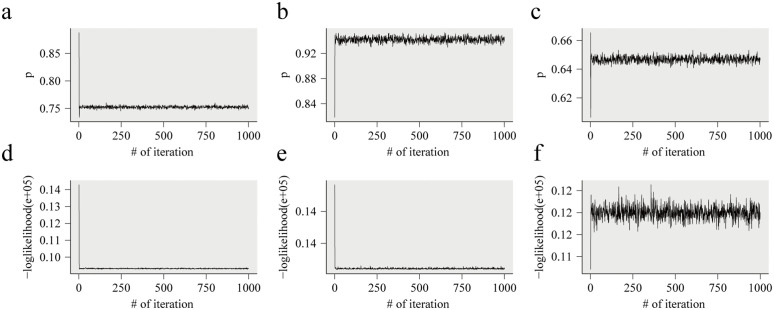
Convergence check trace-plot of simulation experiment results. a and d represent the convergence of bicluster1. b and e represent the convergence of bicluster2. c and f represent the convergence of bicluster3.

To further validate the clustering performance of EBBM on simulated data, a convergence check was performed, as shown in Fig 2. The first row of Fig 2 shows the historical trace plots of the binomial distribution parameters for the three biclusters, while the second row shows the historical trace plots of their likelihood values. As can be seen from the figures, the parameter values and likelihood values of the binomial distribution for the three biclusters identified by EBBM on simulated data fluctuate around their means as the number of iterations increases, without showing any obvious trends or periodicity. Additionally, for each bicluster, its corresponding parameter values and likelihood values converge almost simultaneously, indicating that the algorithm has indeed reached a converged state.

**Fig 3:**
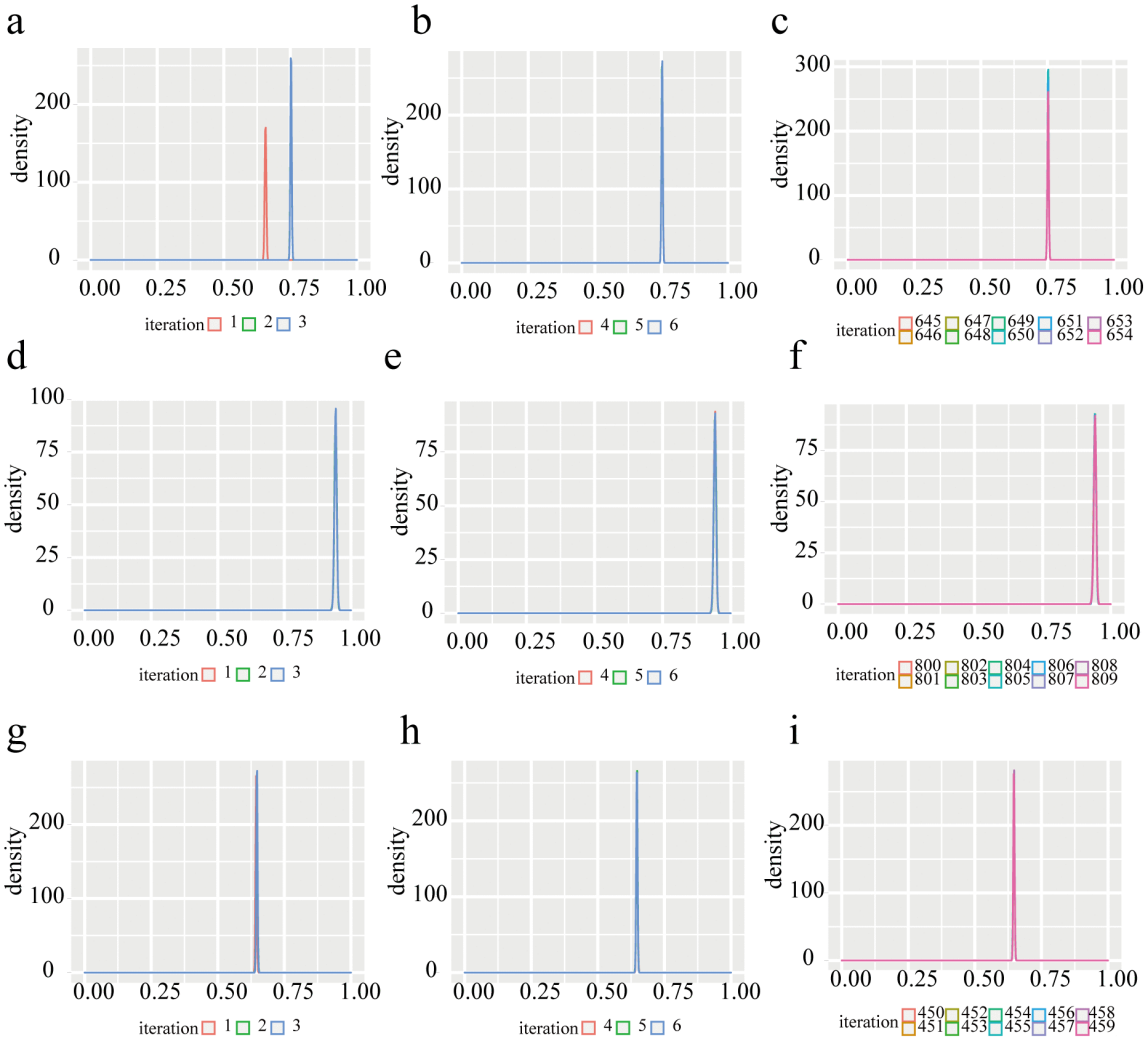
The beta distributions which parameters ρ follow in simulation data experimental result moves with the increase of the number of iterations. a, b, and c represent the movement of the distribution of the parameter corresponding to bicluster1 as the number of iterations increases. d, e, and f represent the movement of the distribution of the parameter corresponding to bicluster2 as the number of iterations increases. g, h, and i represent the movement of the distribution of the parameter corresponding to bicluster3 as the number of iterations increases.

Additionally, the posterior distribution of parameter ρ of the binomial distribution followed by the bicluster is beta-distributed. According to the data screening strategy, as the number of iterations increases, its shape parameters should gradually converge toward the property $a_{i,j}>\text{1}.\text{5}\times b_{i,j}$, meaning that its two shape parameters $\alpha^{\text{bcl}}+\sum_{\left\{i,j|i\in I^{\prime },j\in J^{\prime }\right\}}a_{i,j}$ and $\beta^{\text{bcl}}+\sum_{\left\{i,j|i\in I^{\prime },j\in J^{\prime }\right\}}b_{i,j}$ should gradually satisfy the property $a_{i,j}>\text{1}.\text{5}\times b_{i,j},i\in I^{\prime },j\in J^{\prime }$ during the iteration process. The mean of the beta distribution corresponding to each iteration should also gradually move toward a value greater than 0.6. Therefore, the distribution of this parameter was examined in the experiment as the number of iterations increased. Considering the large total number of iterations, the first six iterations were selected for each of the three biclusters discovered by EBBM, and then 10 iterations were randomly selected to observe the distribution of the corresponding parameters. As shown in Fig 3, it can be seen from the figure that the mean values of the parameter distributions corresponding to the three biclusters all move rapidly toward values greater than 0.6 and quickly converge to their target distributions. As the number of iterations increases, their corresponding distributions almost completely overlap with their target distributions. This indicates that the data screening strategy can indeed guide the parameters of the binomial distribution toward the desired attributes, thereby mitigating the impact of sequencing errors introduced by MeRIP-Seq.

Subsequently, the experiment selected commonly used mainstream biclustering algorithms and applied them to the simulated data, comparing their clustering results with those of EBBM to evaluate the clustering performance of EBBM on the simulated data.

The scoring criteria in the experiment were selected based on commonly used evaluation indicators for biclustering algorithms, namely recovery and relevance scores, as shown in Eq. (1) and (2).


$$\begin{array}{c} F_{1-score}(M_{1},M_{2})=\frac{\text{1}}{\left|M_{\text{1}}\right|}\sum_{A_{i}\in M_{\text{1}}}\underset{B_{j}\in M_{\text{2}}}{max}(F_{\text{1}-score}(A_{i},{\text{B}}_{j})) \end{array}$$ (1)

$$\begin{array}{c} F_{1-score}(M_{\text{2}},{\text{M}}_{\text{1}})=\frac{\text{1}}{\left|M_{\text{2}}\right|}\sum_{B_{j}\in M_{\text{2}}}\underset{A_{i}\in M_{\text{1}}}{max}(F_{\text{1}-score}(B_{j},{\text{A}}_{i})) \end{array}$$ (2)

**Fig 4:**
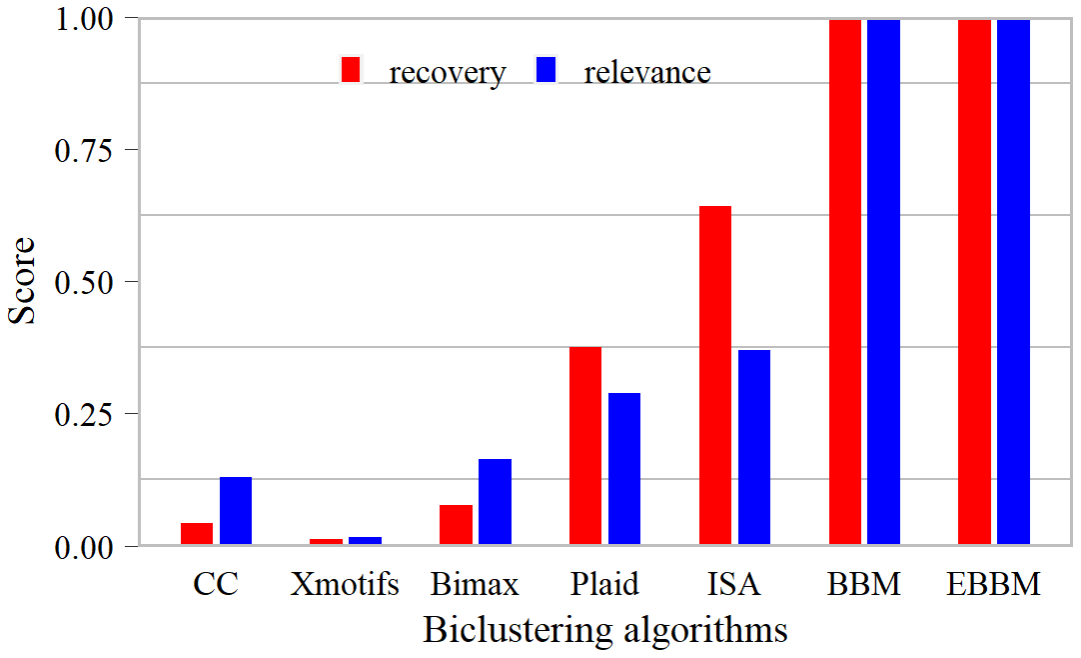
Comparison of clustering results between EBBM and five mainstream biclustering algorithms on simulated data.

Where, $F_{\text{1}-score}(\text{A},\text{B})=\frac{\text{2}(s_{A}\cap s_{B})(c_{A}\cap c_{B})}{n_{A}+n_{B}}s_{A}$ and $s_{B}$ are the numbers of methylation sites in A and B, respectively. $c_{A}$ and $c_{B}$ are the condition numbers in A and B, respectively. $n_{A}=s_{A}\cdot c_{A}$ and $n_{B}=s_{B}\cdot c_{B}$ represent the number of elements in A and B, respectively. A represents the true bicluster, while B represents the predicted bicluster. $M_{\text{1}}=\{A_{\text{1}},...,A_{K}\}$ is the set of true biclusters, and $M_{\text{2}}=\{B_{\text{1}},...,B_{L}\}$ is the set of predicted biclusters. Eq. (1) indicates the model’s ability to recover biclusters, i.e., the recovery score. Eq. (2) indicates the correlation between the model’s predicted biclusters and the true biclusters, i.e., the relevance score. The closer the values of the recovery score and relevance score are to 1, the better the clustering effect. Since mainstream biclustering algorithms all require inputs to be single data matrices, therefore, the traditional computational methods was first employed to convert the simulated IP sample reads count matrix and the input reads count matrix into a single data matrix approximating methylation levels. Then, this matrix was fed into various clustering algorithms for cluster analysis. Finally, the experimental results of EBBM and commonly used mainstream biclustering algorithms are shown in Fig 4. As can be seen from Fig 4, without the addition of simulated data with low methylation levels, the recovery scores and relevance scores of EBBM and BBM algorithms are comparable, but significantly better than the other five biclustering algorithms. This is understandable. First, from a theoretical perspective, the process of generating simulated data is consistent with the modeling principles of EBBM and BBM. In the absence of noise, the data screening strategy of EBBM does not work, so the scores of the two are comparable, while other biclustering algorithms use different algorithmic principles. ISA adopts a threshold optimization strategy, so its clustering performance is relatively better than other algorithms. Secondly, the five biclustering algorithms used in the experiment all used methylation level data as input data. According to traditional calculation methods, methylation level data actually contains noise, but the modeling process of these five methods did not consider noise reduction treatment, so their clustering performance was relatively poor on this simulated data. In addition, the experimental results also indicate that EBBM is more suitable than current mainstream biclustering algorithms for mining m^6^A epigenetic transcriptomics data.

To further evaluate the performance of this data screening strategy in eliminating noise introduced by MeRIP-Seq sequencing errors, it is considered adding noise to the simulated data by embedding a low-expression bilcluster while maintaining its overall distribution characteristics similar to that of the true MeRIP-Seq data.

**Fig 5:**
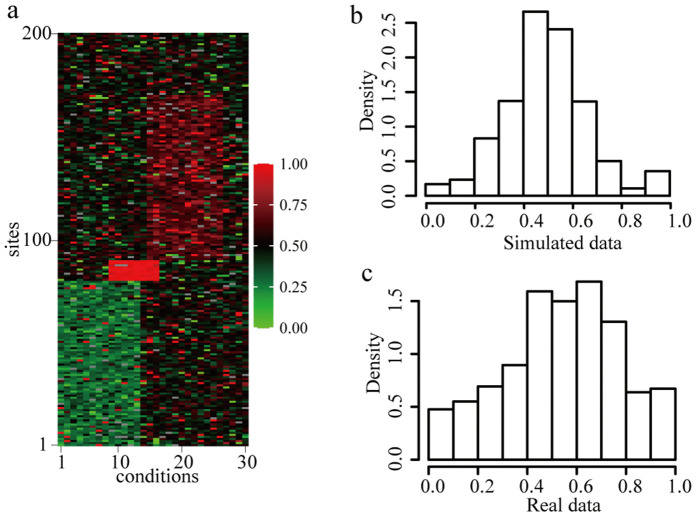
Comparison of Statistical characteristics between simulated data and real data. a Heatmap of simulated methylation level data. b Histogram of simulated methylation level data. c Histogram of real methylation level data.

To this end, the experiment simulated the reads count data of IP samples and input samples at 200 sites across 30 experimental conditions. Three biclusters are embedded in the data, with sizes of 80×13, 10×8, and 80×12, respectively, and the remaining part is background. The parameters ρ of their binomial distributions are 0.3, 0.98, 0.66, and 0.5, respectively. Among them, the bicluster with ρ = 0.3 is the embedded noise data. The three biclusters do not overlap in site. In terms of conditions, the first two biclusters have five conditions overlap, while the second and third biclusters have three conditions overlap. The heat map of the converted methylation level data, the simulation data histogram, and the comparison of the real data histogram are shown in Fig 5.

Subsequently, the above simulated data was fed into EBBM, with the initial iteration count set to 1000, the burn-in count set to 500, the predefined number of biclusters set to 10, and the intra-chain variance threshold set to 0.1. Finally, EBBM output two biclusters. The first bicluster contained 10 sites, and the second bicluster contained 80 sites. In terms of conditions, the first bicluster contained 8 conditions, and the second bicluster contained 12 conditions. Both bicluster 1 and bicluster 2 contained sites and conditions that were completely consistent with the actual situation. Additionally, the two overlap conditions in bicluster 1 and bicluster 2 were accurately reproduced.

**Fig 6:**
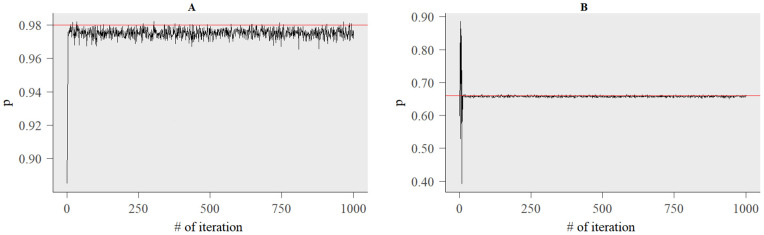
Trace-plot of parameter ρ of EBBM clustering result on simulated data.

When examining the relevant parameters of the two biclusters and the elements they contain, it was found that the mean value of parameter ρ for the binomial distribution corresponding to the first bicluster is 0.9749736. The mean value of parameter ρ for the binomial distribution corresponding to the second bicluster is 0.6580733. Both values all approach the ground-truth. Their changes with increasing iteration counts are shown in Fig 6. Fig 6 indicates that as the iteration count increases, the two parameter values fluctuate steadily within a certain range, showing no obvious trend or periodicity, which suggests that the algorithm has converged.

**Fig 7:**
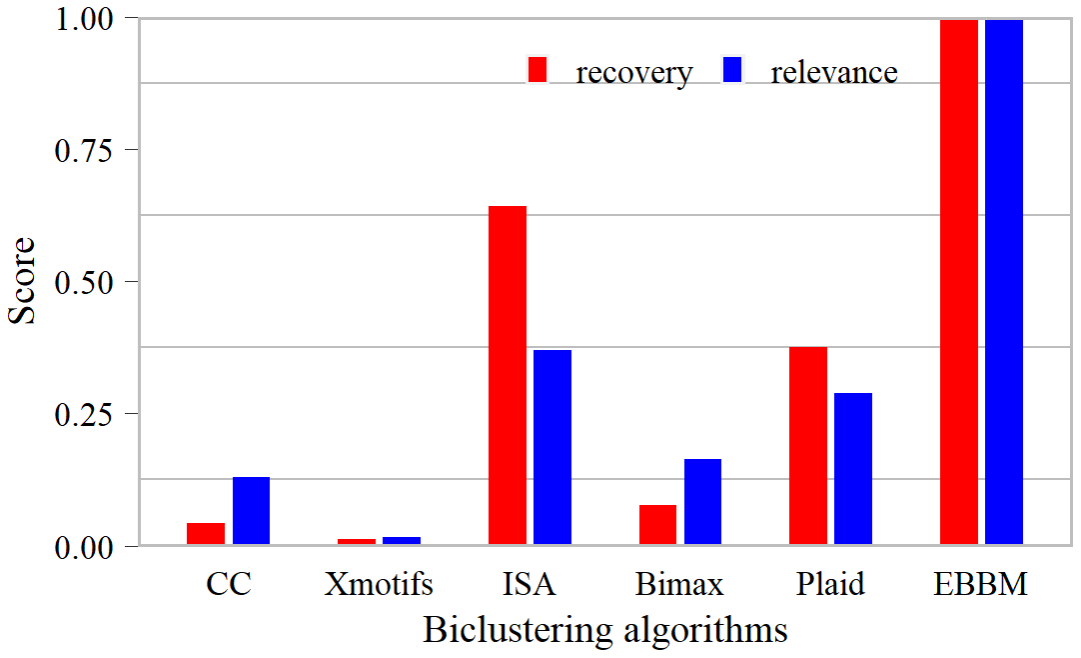
Comparison of clustering results of EBBM, BBM and other 5 mainstream biclustering algorithms on simulated data with noise.

Additionally, the recovery and relevance scores were examined in the experiment, as shown in Fig 7. Fig 7 shows that when noise is added, the recovery and relevance scores of EBBM are 0.998 and 0.997, respectively, with performance nearly equivalent to that on simulated data without noise. However, BBM’s recovery and relevance scores dropped significantly on noisy data, reaching 0.66 and 0.67, respectively, which are 0.338 and 0.327 lower than EBBM’s scores. This indicates that BBM’s performance in noise removal is far inferior to EBBM’s, and EBBM’s data screening strategy is indeed effective in removing noise on simulated data.

### 2.2. Real data experimental analysis

High-precision datasets are a prerequisite for reliable results. Our previous research have construct a data set of 69,446 sites under 32 conditions, based on 6 single-base resolution mi-CLIP and m6A-CLIP experimental and 32 MeRIP-Seq experimental [64], and the majority (about 80.66%) of our sites are supported by at least one of the two high-confidence reference databases, GLORI [71] and m6A‑Atlas v2.0 [72]. This data set is also utilized in this study. The related material details to the data are shown in Table 1.

**Table 1:** Data Sets Used In This Study.

ID	GEO accession Cell line		Treatment	Source
1-4	SRR456542-SRR456549, SRR456551-SRR456557	HepG2	UV, HGF, IFN, UT	[73]
5-6	SRR903368-SRR903379	U2OS	CTL, DAA	[74]
7–10	SRR847358-SRR847377	HeLa	Ctrl, METTL14-, METTL3-, WTAP-	[75]
11–12	SRR1182582-SRR1182590	ES/NPC	hNPC, hESC	[76]
13–18	SRR1182591-SRR1182596 SRR494613-SRR494618, SRR5080301-SRR50312	Hek293T, Hek293A	Ctrl, WTAP-, METTL3-, METTL16-	
19–21	SRR1182597-SRR1182602	OKMS	D0, D5_WITH_DOX, D5_WO_DOX	
22–26	SRR1182603-SRR1182630	A549	Ctrl, METTL14-, METTL3-, WTAP-, KIAA1429-	
27–28	SRR3066062-SRR3066069	AML	Ctrl, FTO+	[77]
29–30	SRR5239086-SRR5239109	AML2	Ctrl, METTL3-	[78]
31–32	SRR1035213-SRR1035224	ESC	T0, T48	[79]

**Fig 8:**
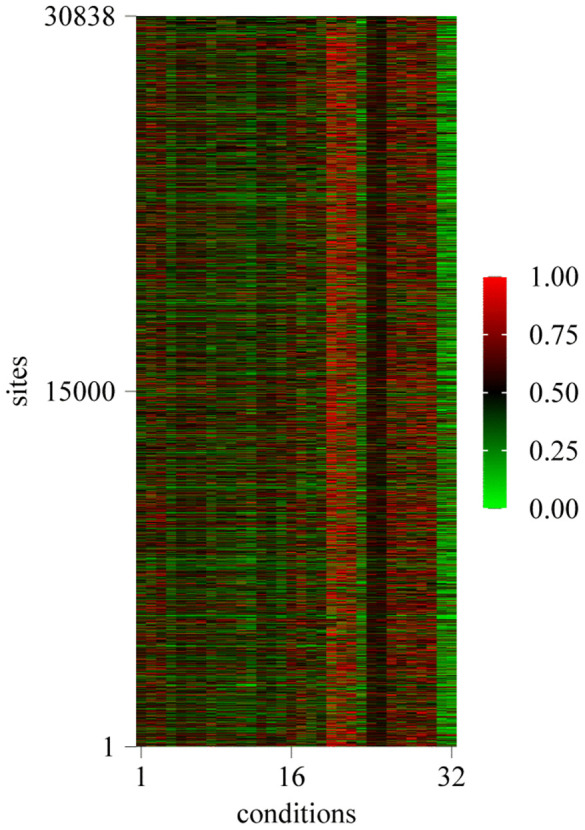
Heatmap of the remained sites across the 32 conditions after variance screening.

All the orginal MeRIP-Seq data are downed form the GEO database. In this study, we followed the method of [80] to quantify the raw data. All the raw data were aligned to the human reference genome of hg19 with Tophat2 [81] to generate the BAM file; then, R script we wrote was run to get the number of reads at each site, normalized to the Fragments Per Kilobase of Transcript Per Million (FPKM) statistics of the site. Since sites with smaller variance under different experimental conditions exhibit more stable methylation modifications under these conditions, meaning that these sites are essentially unaffected by regulatory factors, they cannot be considered as candidate sites for co-methylation patterns. Therefore, variance screening must be performed on m^6^A methylation level data in experimental designs using real data. By visualizing and analyzing the variance distribution of methylation levels at different experimental conditions, it was found that the variance distribution of the sites ranged from 0 to 0.15, with the majority concentrated between 0 and 0.05. To remove low-variance noise sites while preserving most of the sites, different thresholds were tested for variance screening in the experiment. It was ultimately found that when the threshold was set to 0.03, low-variance noise sites were removed while retaining most of the site information. After screening, ultimately, 30,838 index of sites were obtained, and then the IP reads count matrix and input reads count matrix for 30,838 points were obtained. The heatmap of methylation level of 30838 sites across 32 conditional samples as shown in Fig 8.

**Fig 9:**
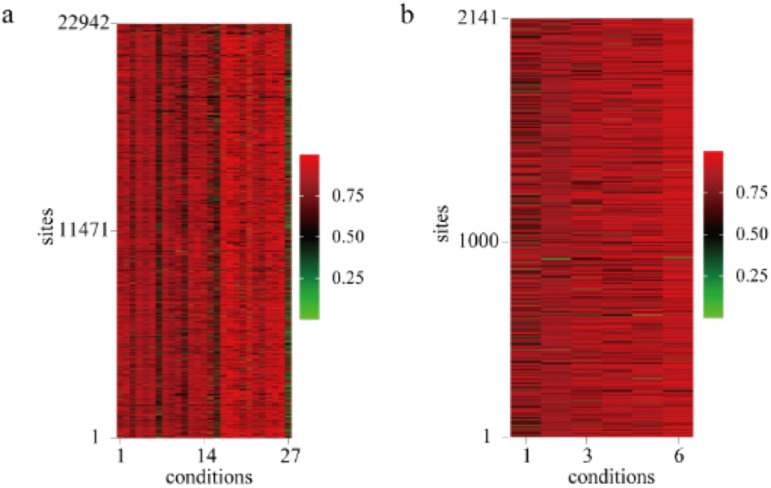
Heatmap of methylation level data of EBBM clustering results on real data.

The above two matrices was then fed into the EBBM model, the number of initial iterations was set to 1,500, the number of burn-in iterations to 500, the intra-chain variance to 1e-06, and the number of initial biclusters to 15. Finally, the model output two patterns containing 22,942 and 2,141 sites, respectively, with condition numbers of 27 and 6. When these two patterns were visualized as methylation levels, the data heatmap is shown in Fig 9.

Fig 9 shows that the average methylation levels of the two clusters identified by EBBM in the real data are 0.79 and 0.82, respectively. These values are significantly higher than the methylation levels of the clustering results without data screening strategies. This indicates that this method can effectively remove noise introduced by errors in the MeRIP-Seq sequencing technology. However, Fig 9 also shows that the methylation levels of the sites contained in columns 6, 9, 16, and 27 are significantly low. This is because, during the modeling process, only the sites were screened, while no data screening strategy was applied to the conditions. This approach was chosen primarily because there were relatively few conditions, and secondly, if both sites and conditions were subjected to data screening strategies, the model would easily reach a local optimum, resulting in the loss of most sites. Experimental results showed that when both sites and conditions were subjected to data screening strategies on simulated data, the results were also poor.

1) Pathway specificity analysis

In order to further verify whether the clustering results of the EBBM model on real data are valid local co-methylation patterns, six pathways known to be significantly associated with m^6^A were first selected for pathway correlation analysis. The Fisher’s exact test and multiple hypothesis testing methods were used to analyze whether the sites retained in the clustering results were significantly associated with known pathways. The significance level was set at 0.01. The enrichment results are shown in Table 2.

**Table 2:** Pathway-specific analysis of EBBM clustering results on real data.

ID	# of sites	Enrichment Statistics	KEGG Pathways					
			Apoptosis	DNA Repair	Fatty Acid Metabolism	P53 Pathway	UV response Down	UV response Up
P1	22942	OR	1.08	0.68	0.41	0.99	2.18	0.68
		p-value	0.71	0.04	**1.74e-05**	1	**5.93e-04**	0.05
		FDR	0.85	0.08	**1.04e-04**	1	**1.78e-03**	0.08
P2	2141	OR	1.39	1.09	1.57	1.04	1.87	1.11
		p-value	0.18	0.78	0.09	0.81	0.01	0.67
		FDR	0.35	0.81	0.27	0.81	0.07	0.81

Table 2 shows that, after multiple hypothesis testing, Pattern 1 is significantly correlated with fatty acid metabolism and UV response down. When the METTL3 gene is silenced, fatty acid metabolism can cause a decrease in m^6^A methylation and total mRNA levels of fatty acid synthesis. At the same time, when METTL3 is silenced and there is no METTL3 catalytic activity, cells show delayed repair of UV-induced cyclobutane pyrimidine adducts and increased sensitivity to UV radiation [82]. Table 2 indicates that pattern1 may be significantly associated with pathways related to reduced m^6^A modification caused by METTL3

gene silencing. Pattern2 was not enriched in the aforementioned known pathways associated with m^6^A. Therefore, we began to search for other biological explanations to explore the biological significance of Pattern2 at other levels. Existing studies have shown that m^6^A methylation is influenced by enzyme regulation, so we then analyzed the substrate specificity of the enzyme.

2) Analysis of enzyme substrate specificity

Firstly, 12,643 METTL3, 7,689 METTL14, 13,124 WTAP, 399 KIAA1429, and 10,030 FTO enzyme target sites were obtained from an independent public study [75]. These target sites were obtained by knocking out the relevant enzyme genes, acquiring raw data, and then mapping them to the human hg19 genome using exomePeak, followed by extracting RNA enzyme target methylation sites with significantly low expression (p-value < 0.05). Secondly, the gene symbols and Entrez Gene IDs corresponding to the sites included in the two patterns discovered by EBBM were annotated. Subsequently, Fisher’s exact test and multiple hypothesis testing methods were used to explore the enrichment relationship between the two biclusters discovered by EBBM and the target sites of enzymes. In the enrichment analysis, the significance level was set to 0.05, and the BH method [83] was used for multiple hypothesis testing. The final enrichment results are shown in Table 3.

**Table 3:** Enzyme-specificity analysis of EBBM clustering results on real data.

ID	# of sites	Enrichment Statistics	Methyltransferase Component				
			METTL3	METTL14	WTAP	KIAA1429	FTO
P1	22942	OR	0.63	0.23	0.41	0.83	1.07
		p-value	**7.96e-176**	**0**	**0**	**4.74e-04**	0.28
		FDR	**1.33e-175**	**0**	**0**	**5.92e-04**	0.28
P2	2141	OR	0.37	0.02	0.05	0.44	0.99
		p-value	**1.74e-105**	**1.03e-266**	**0**	**5.69e-06**	1
		FDR	**1.74e-105**	**1.03e-266**	**0**	**5.69e-06**	1

Table 3 shows that, after multiple hypothesis testing, Pattern1 and Pattern2 were significantly enriched in the specific target sites of four methyltransferase complexes, METTL3, METTL14, WTAP, and KIAA1429, respectively, but the degree of enrichment was significantly different. This indicates that the m^6^A sites retained in Pattern1 and Pattern2 are regulated to varying degrees by methyltransferases. In pathway analysis, Pattern2 was not significantly associated with known pathways significantly regulated by m^6^A, but it was significantly enriched in specific target sites of methyltransferases, indicating that this pattern is also an effective local co-methylation pattern.

The above analysis only demonstrates the significant correlation between the two patterns discovered by EBBM in real data and the known research results related to m^6^A methylation modification. In order to further explore the other biological significance of the two patterns discovered by EBBM, GO enrichment analysis was subsequently performed.

3) GO enrichment analysis

**Fig 10:**
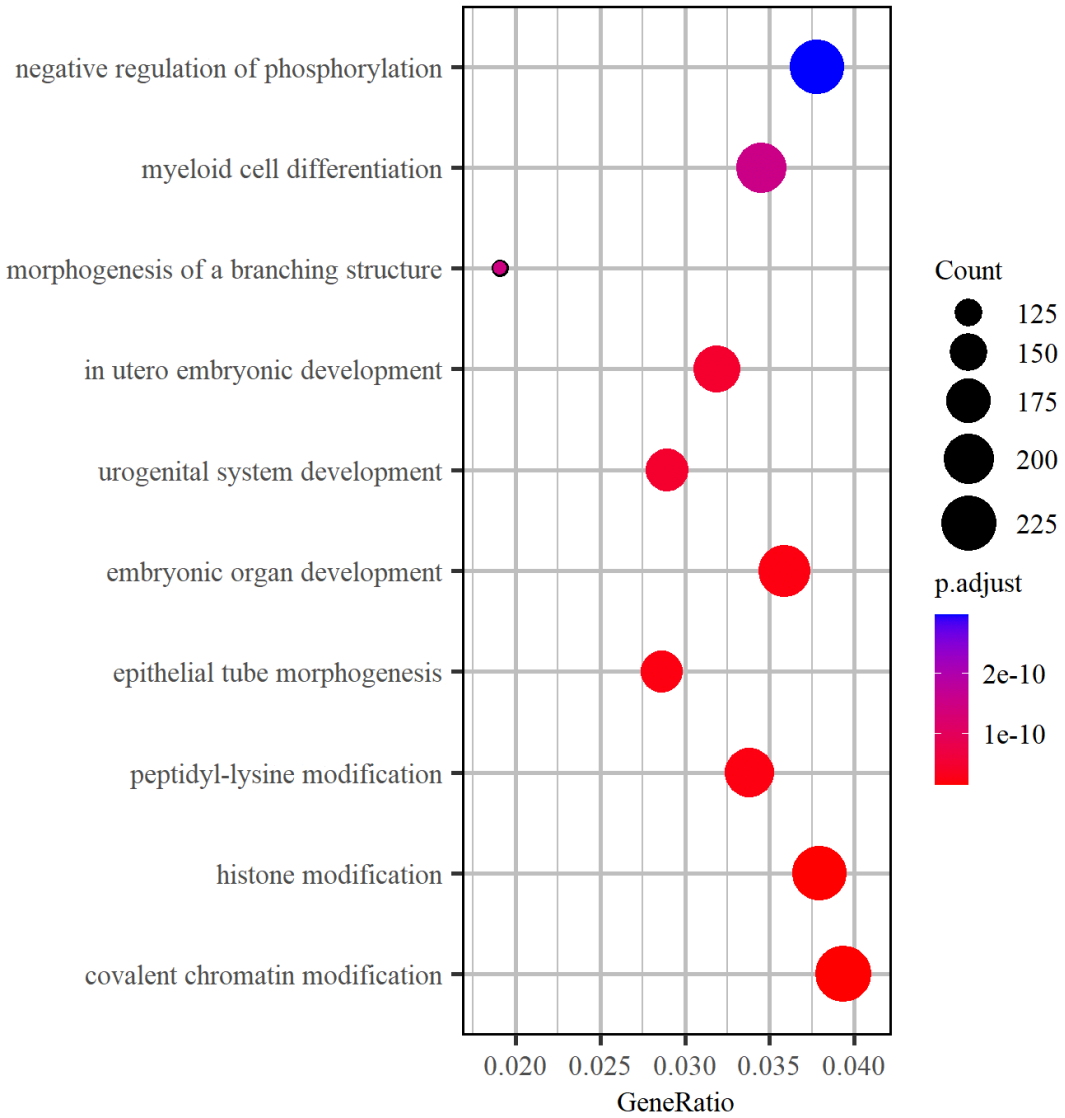
Enrichment results of the sites retained in Pattern1 on BP term.

**Fig 11:**
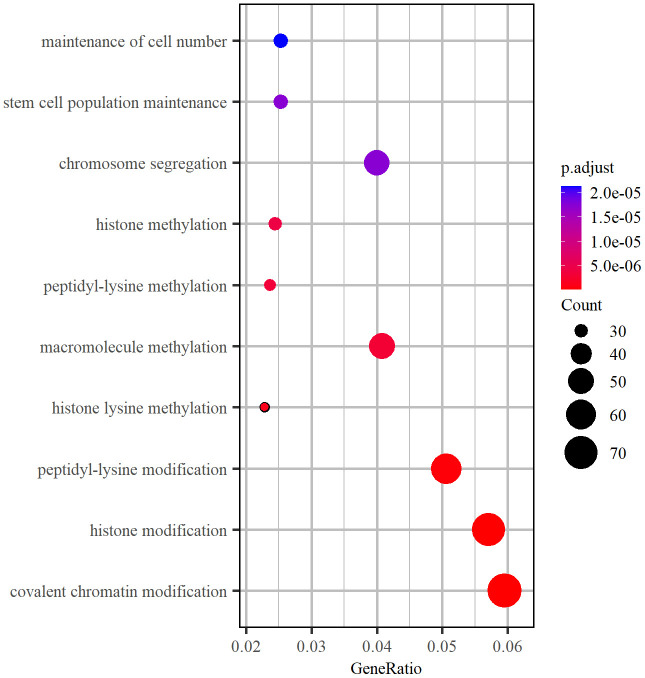
Enrichment results of the sites retained in Pattern2 on BP term.

In the GO enrichment analysis, the Gene Symbol and Entrez Gene ID corresponding to the genes retained in pattern1 and pattern2 were first annotated, and then Fisher’s exact test was used to explore the biological terms of the biological processes enriched in the gene ontology of the sites retained in pattern1 and pattern2. When retaining the top 10 most enriched BP terms in pattern1 and pattern2, their enrichment results are shown in Figs 10 and 11. Fig 10 indicates that pattern1 is associated with biological processes such as negative regulation of phosphorylation, differentiation of bone marrow cells, and embryonic stem cell development [84], which is consistent with previous studies. Fig 11 shows that pattern2 is associated with biological processes such as peptide lysine methylation and chromosome separation [85], which is consistent with previous wet lab-related studies.

**Fig 12:**
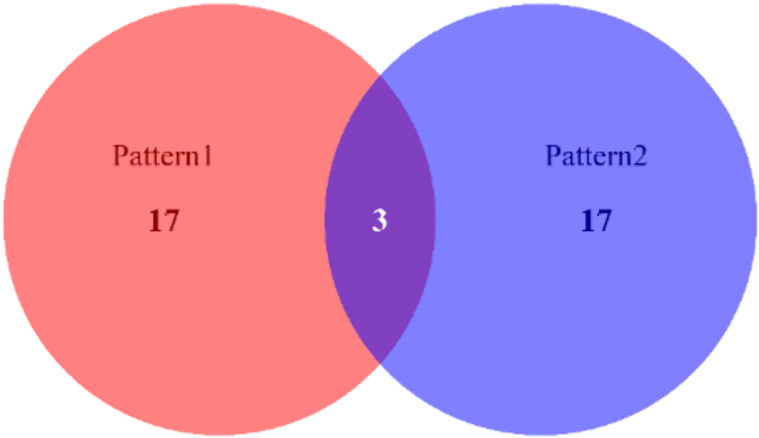
The overlap of the top 20 enrichment BP terms by the sites remained in Pattern1 and Pattern2.

In addition, Figs 10 and 11 show that pattern1 and pattern2 are both related to biological processes such as histone modification, covalent chromatin modification, and peptidyl lysine modification. Therefore, the overlap of the top 20 BP terms most enriched in pattern1 and pattern2 was examined, as shown in Fig 12. Fig 12 shows that although there are three overlaps among the top 20 most enriched BP terms retained in the BP GO enrichment results of pattern1 and pattern2, most of the enriched BP terms are still different for each pattern. For example, pattern1 is enriched in intracellular receptor signaling pathways, protein acylation, gland development, kidney development, etc., while pattern2 is enriched in protein methylation, protein alkylation, mitosis and meiosis, RNA splicing, etc. The above analysis shows that the clustering results of EBBM on real data are condition-specific to a certain extent.

To further illustrate the clustering performance of EBBM on real data, the GOE_Score scoring standard was finally selected, as shown in Eq. 3 [58].


$$\begin{array}{c} \text{GOE}\_\text{Score}=\frac{g_{t\text{1}}+g_{t\text{2}}+...+g_{tT}}{g_{lcp}} \end{array}$$ (3)

In Eq. (3), $g_{t\text{1}}$ represents the number of genes contained in term *t*_1_ that is significantly enriched in *t*he local co-methylation pattern. *T* represents the total number of terms enriched in this local co-methylation pattern. $g_{lcp}$ represents the total number of genes contained in this local co-methylation pattern. The higher the score for GOE_Score, the more biologically significant the local co-methylation pattern discovered.

**Fig 13:**
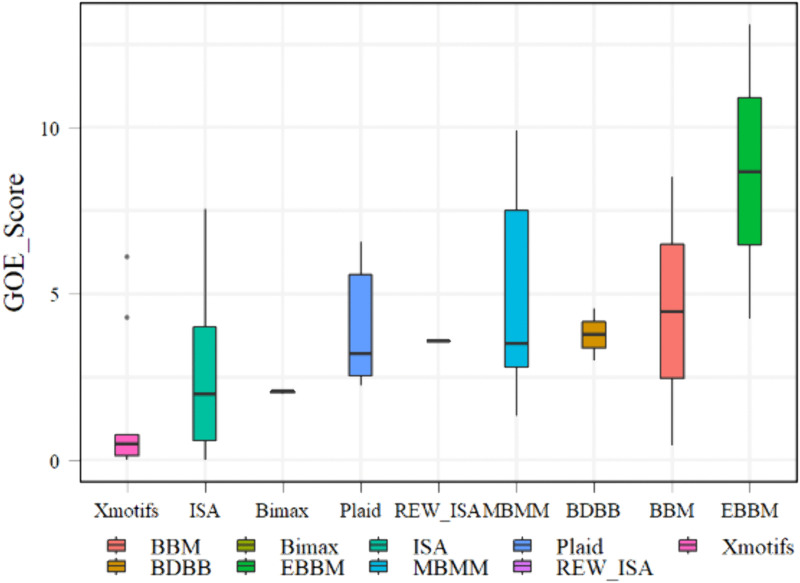
Comparison of GOE_Score between EBBM and current mainstream biclustering algorithm on real data.

In the aforementioned comparative experiments, since the real data used were IP sample reads count data and input sample reads count data, while mainstream biclustering algorithms require the input data to be a single data matrix. Therefore, to compare the performance of EBBM and other mainstream algorithms, the aforementioned lossless data was first normalized into a FPKM value data matrix, and then further converted into a methylation level matrix describing m^6^A methylation modification using the traditional method for calculating methylation levels. Finally, the methylation level matrix data was fed into each mainstream biclustering algorithm, and their GOE_Score scores were calculated based on their clustering results and Eq. (3). For the BBM and EBBM models, the IP sample reads count data and input reads count data were directly input. In addition, the Xmotifs, ISA, Bimax, and Plaid algorithms in the experiment all use the parameters recommended in the relevant literature [86]. The GO enrichment analysis term was set to ALL, which includes CC, BP, and MF. The comparison results are shown in Fig 13. Fig 13 shows that EBBM’s clustering performance on real data is significantly better than other biclustering algorithms, and the average GOE_Score score of the local co-methylation patterns it discovers is significantly higher than that of the BBM model. This suggests that the biclusters discovered by EBBM in real MeRIP-Seq data are more biologically meaningful than the other biclustering algorithms scored lower primarily because the methylation level data was distorted and noisy, and secondly because these biclustering methods did not take measures to penalize noisy data. For BBM, although its model is based on distortion-free IP and input data, it has overcome some of the noise issues introduced during data preprocessing, such as normalizing reads count data to FPKM values. However, due to the sequencing noise inherent in the MeRIP-Seq sequencing technology, this issue has not been addressed in the BBM model. Therefore, in theory, the GOE_Score scores of its clustering results should be lower than those of EBBM. The results in Fig 13 further validate the rationality of the aforementioned analysis.

4) Data sensitivity analysis on threshold selection

To demonstrate the stability of the results with respect to threshold selection, we performed a data sensitivity analysis on threshold selection.

Set the analysis range, re-evaluate key results (such as number of retained sites after screening, average bicluster size, overlap of identified co-methylation patterns (Jaccard similarity), biological consistency of top enriched pathways (GO/KEGG) for biclusters obtained under each threshold.) at different IP/Input ratios, including 1.0, 1.2, 1.4, 1.5, 1.6, 1.8, 2.0 and 2.5. Experimental results (S1 Fig) showed that the number of retained sites decreased as the ratio increased, but the core co-methylation patterns (i.e., biclusters with high stability) remained largely unchanged when the ratio ranged from 1.2 to 2.0. The Jaccard similarity between biclusters obtained at 1.5 and those at 1.8 was > 0.85. Importantly, the top enriched biological processes (e.g., “Histone modification”, “peptidyl-lysine modification”) were consistently reproduced across thresholds from 1.2 to 2.0. When the ratio was set to 1.0 (i.e., no strict filtering) or 2.5 (overly stringent), the biclustering results became unstable or lost meaningful patterns.

The above indicates that the 1.5 threshold is a reasonable default choice, and our algorithm’s performance is robust to moderate variations (±0.3) around this value.

## 3. Discussion

The exploration of co-methylation patterns based on m^6^A sequencing data can assist biological experiments in studying their functional mechanisms, saving time and economic costs, and thereby exploring the possibility of treating complex diseases such as cancer from the perspective of the epigenetic transcriptome. However, due to the inherent complexity of MeRIP-Seq sequencing data, particularly the inherent sequencing errors associated with this sequencing technology, a certain number of false-positive data points are present in the sequencing data. For example, data where the count of reads for the IP sample at a given site is less than 1.5 times that of the input sample is typically attributed to sequencing errors. Such data has a low signal-to-noise ratio. Currently, mainstream co-methylation detection algorithms in this field generally suffer from issues such as insufficient robustness, low accuracy, and unreliable clustering results.

To address this issue, this paper proposes a beta-binomial distribution biclustering algorithm based on data screening strategies, EBBM. This algorithm improves the robustness of the model to low signal-to-noise ratio data by introducing data screening strategies into the beta-binomial distribution modeling process, thereby improving the reliability of the clustering results. Inspired by current retrieval-augmented generation technique, this algorithm creatively introduces a data screening strategy in the process of constructing models using Bayesian methods, successfully guiding the data flow toward areas with high signal-to-noise ratios, so that the discovered patterns contain sites that are mostly actually methylated. Simulation data experiments show that the EBBM algorithm can effectively mine local co-methylation patterns pre-set in the data. On data without low signal-to-noise ratio (SNR) implantation, the F1 evaluation index shows that EBBM and the current state-of-the-art algorithm BBM are basically equivalent. However, after implanting low SNR data, EBBM scores significantly higher than BBM. In real m^6^A sequencing data, EBBM discovered two effective co-methylation patterns, which were enriched in negative regulation of phosphorylation and peptidyl lysine methylation, etc. different biological processes. Analysis of m^6^A pathway specificity and enzyme substrate specificity indicated that this pattern was an effective co-methylation pattern. In the GO enrichment analysis, the two patterns were enriched in different biological terms, indicating that they have certain condition specificity in their functional patterns. At the same time, there is a small overlap between the two patterns in GO terms. Combined with their pathway analysis, it was found that these two patterns may be regulated by the same regulatory factors to a certain extent, but the regulatory intensity is significantly different. Finally, the GOE_Score scoring results indicate that the co-methylation patterns mined by EBBM are more biologically meaningful than the mining results of current mainstream algorithms.

It is worth noting that more recent probabilistic or deep learning-based biclustering methods were not included as baselines in this study. A review of the literature indicates that such methods—developed primarily for other omics data—are not suitable for direct comparison with our approach. For instance, among probabilistic biclustering algorithms, BGB [87] is designed for a single data matrix in which rows represent independent features. While this makes BGB applicable to conventional omics data, it cannot accommodate the paired-matrix structure of MeRIP-seq data, which consists of two matched matrices (IP and input) measuring the same 65,536 modification sites across 32 conditions. The biological quantity of interest is the enrichment contrast between IP and input, which BGB cannot explicitly model. Any artificial data transformation to force compatibility would yield biclustering results that are not interpretable as differences between IP-enriched and non-enriched sample groups, rendering BGB scientifically untenable as a baseline. Among deep learning-based biclustering algorithms, autoencoder-driven and GNN-based methods have been proposed. A representative autoencoder-driven method, scDBic [88], presents three major issues when applied to MeRIP-seq data. First, it selects “key genes” with low within-cluster expression variance, whereas MeRIP-seq requires sites exhibiting high variance in enrichment contrast. Second, its 128-dimensional bottleneck is invalid for our dataset comprising only 32 samples. Third, it cannot handle two paired measurements (IP and input): using only IP omits essential background correction, while using the IP/input ratio violates its non‑negative integer count assumption. Consequently, scDBic is excluded due to a mismatch in biological targets, scalability constraints, and input format incompatibility. As a representative GNN-based biclustering method, Gaebic [89] requires a precomputed single-matrix feature–sample correlation graph as input. However, our MeRIP-seq data consist of two matched matrices per sample, and no public implementation supports this paired layout. Moreover, Gaebic is specifically designed for binary (0/1) matrices representing miRNA–gene targeting relationships, where only elements equal to 1 can be modeled. Owing to both dual-input incompatibility and binary-data constraints, Gaebic is also unsuitable as a baseline for our study. For similar reasons above, several recently proposed clustering algorithms [90–92] are likewise inappropriate as baselines for comparison.

The algorithm proposed in this paper represents an innovative modeling approach, providing a reference for constructing models for similar data or application scenarios. Additionally, the data screening strategy employed effectively eliminates noise issues caused by MeRIP-Seq sequencing errors, offering a powerful computational tool for identifying m^6^A co-methylation patterns and studying their functional mechanisms. Furthermore, EBBM identified two effective local co-methylation patterns in real m^6^A sequencing data. These patterns exhibit greater biological significance than the clustering results of current mainstream algorithms, and this finding can serve as a reference for biological experiments investigating m^6^A mechanisms. Experimental analysis shows that EBBM can effectively remove the influence of low signal-to-noise ratio in sequencing data, thereby improving the reliability of clustering results. However, this study also has certain limitations. First, the dataset is not large enough. A dataset that accommodates more experimental samples can further improve the reliability of the clustering results. In addition, it is also found in the experiment that due to the limitations of the experimental sample conditions, the data screening strategy was only performed based on the site during the modeling process. Performing the data screening strategy in multiple dimensions may yield more reliable clustering results. Therefore, in future work, we will further expand the m^6^A dataset to include more experimental samples. We will also attempt to implement data screening strategies simultaneously in two dimensions—site and experimental conditions, to further improve the reliability of the clustering results.

## 4. Methods

### 4.1. Probabilistic graphical model of EBBM

MeRIP-Seq sequencing uses IP samples and input samples to describe the distribution of m^6^A modifications. Based on the purpose of model construction, in this paper, we use $(a_{i,j}{)}_{n\times m}$ denote the reads count data of IP samples and $(b_{i,j}{)}_{n\times m}$ denote the reads count data of input samples, where *n* denotes the total number of sites and *m* denotes the total number of sample conditions. $a_{i,j}$ denotes the number of reads count of IP samples for site *i* under condition *j*, and $b_{i,j}$ denotes the number of reads count of input samples for site *i* under condition *j.*

In order to represent the degree of methylation modification of m^6^A under different conditions, the traditional method is described by calculating the methylation level as shown in Eq. (4).


$$\begin{array}{c} l_{i,j}=\frac{a_{i,j}}{a_{i,j}+b_{i,j}} \end{array}$$ (4)

$l_{i,j}$ denotes the methylation level of site *i* under condition *j*. Now, the following transformation is made to (4), which therefore leads to (5).


$$\begin{array}{c} a_{i,j}=l_{i,j}(a_{i,j}+b_{i,j}) \end{array}$$ (5)

According to Eq. (4), $\text{0}<l_{i,j}<\text{1}$, and hence, according to the expectation $E[X]=\rho (a_{i,j}+b_{i,j})$ of the binomial distribution, it follows that (5) can be regarded as the expectation of the $a_{i,j}\sim \text{Bi}\text{nomial}(a_{i,j}+b_{i,j},\rho )$.

Where $\rho =l_{i,j}$ denotes the probability of success in each experiment. $a_{i,j}+b_{i,j}$ denotes the number of Bernoulli experiments performed in each binomial distribution experiment, which $a_{i,j}$ can be viewed as a random sample from a binomial distribution with parameters $\left(a_{i,j}+b_{i,j},\rho \right)$.

By the expectation of the binomial distribution, it follows that $a_{i,j}$ is sampled with a large probability of converging to its expected value, that is $a_{i,j}\approx \rho (a_{i,j}+b_{i,j})$.

Therefore, it is reasonable to assume that the number of IP reads count of site *i* under condition *j* follows a binomial distribution with parameter as shown in Eq. (6).


$$\begin{array}{c} a_{i,j}\sim \text{Binomial}(a_{i,j}+b_{i,j},\rho ) \end{array}$$ (6)

$a_{i,j}+b_{i,j}$ denotes the number of Bernoulli experiments performed in each binomial distribution experiment, and ρ denotes the probability of a positive case in each binomial distribution experiment. $a_{i,j}$ can be viewed as a random sample from the binomial distribution with parameter $\left(a_{i,j}+b_{i,j},\rho \right)$.

**Fig 14:**
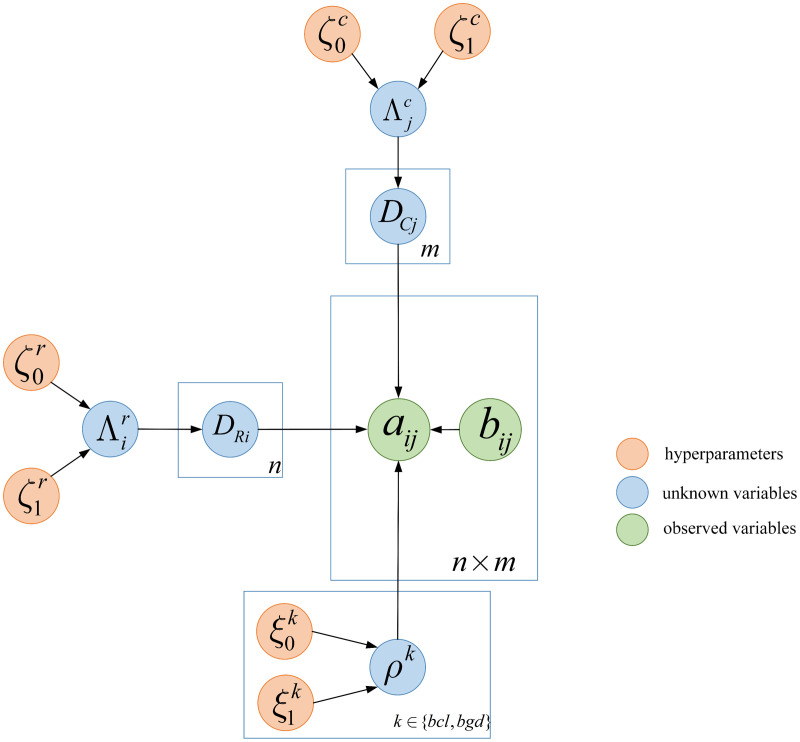
Probabilistic graphical model of BBM.

Therefore, the data of the whole IP sample can be viewed as consisting of c,c=1,...,K local co-methylation patterns and 1 background data, each co-methylation pattern respectively follows a relatively sharp binomial distribution with parameter $p=\rho_{c}^{\text{blc}}$, and the background part follows another relatively flat binomial distribution with parameter $\rho^{\text{bgd}}$. This Beta- binomial mixing (BBM) model can be described by the probability graphical model, which is represented as shown in Fig 14.

In Fig 14, $D_{R}=(D_{R\text{1}},D_{R\text{2}},...,D_{Rn})$ and $D_{c}=(D_{C\text{1}},D_{C\text{2}},...,D_{Cm})$ are vectors of indicator variables for sites (rows) and conditions (columns), respectively, and $D_{Ri}=\text{1},i=\text{1},\text{2},...,n$ denotes that site *i* belongs to the bicluster, otherwise it belongs to the background. $D_{Cj}=\text{1},j=\text{1},\text{2},...,m$ denotes that condition *j* belongs to the bicluster, otherwise it belongs to the background. $D_{Ri},i=\text{1},\text{2},...,n$ and $D_{Cj},j=\text{1},\text{2},...,m$ are samples from Bernoulli distributions with parameters $\Lambda_{i}^{r}$ and $\Lambda_{j}^{c}$, respectively, and $\rho^{k}$ are parameters of the corresponding binomial distributions. $\left(\zeta_{\text{0}}^{r},\zeta_{\text{1}}^{r}\right)$, $\left(\zeta_{\text{0}}^{c},\zeta_{\text{1}}^{c}\right)$, $\left(\xi_{\text{0}}^{k},\xi_{\text{1}}^{k}\right)$ are the hyperparameters of the prior distributions of $\Lambda_{i}^{r}$, $\Lambda_{j}^{c}$, $\rho^{k}$, i.e., the shape parameters of the corresponding beta distributions.

Therefore, the data generation process can be viewed as follows: first, $\Lambda_{i}^{r}$ and $\Lambda_{j}^{c}$ are generated from the beta distributions, respectively, and then the Bernoulli distributions generate the labels $D_{R}=(D_{R\text{1}},D_{R\text{2}},...,D_{Rn})$ and $D_{c}=(D_{C\text{1}},D_{C\text{2}},...,D_{Cm})$ for each site and condition, according to which the data indexed by them are determined to belong to the bicluster or the background, and the corresponding binomial distributions are chosen to generate the corresponding observations.

The above model can mine the co-methylation patterns hidden in the data, however, due to the existence of sequencing errors inherent in MeRIP-Seq technology, usually, the sequencing values with the number of IP reads count at a site less than 1.5 times of the number of input reads count at that site are regarded as sequencing noises, and the BBM could not distinguish such noises efficiently, therefore, the above model needs to be further adapted to improve its robustness to these low signal-to-noise data and enhance the reliability of the clustering results.

**Fig 15:**
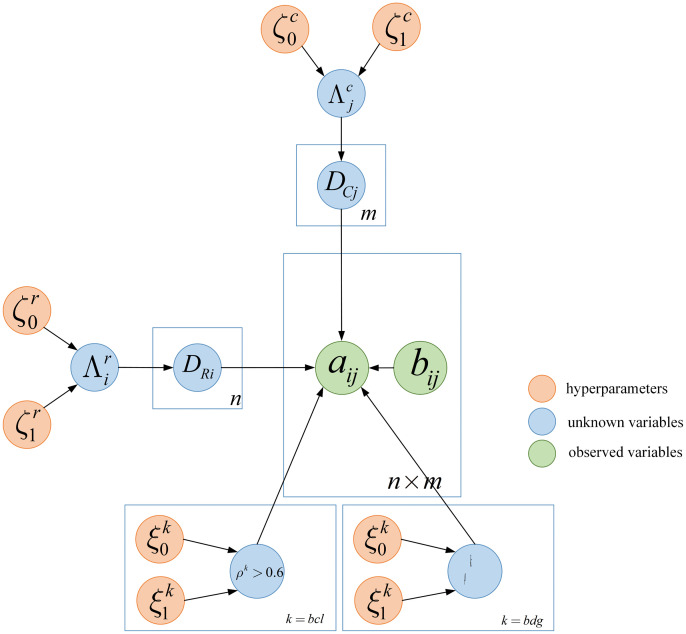
Probabilistic graphical model of EBBM.

The Retrieval-augmented Generation technique improves the reliability of the large model by providing it with information retrieved from specific data sources as a means to correct and supplement the generated answers. Inspired by this, this paper effectively removes the effect of MeRIP-Seq sequencing errors and improves the reliability of clustering results by introducing a data screening strategy based on the BBM model. That is, when the data classification is determined by the initial label, and then the related binomial distribution is determined to generate the corresponding observation data, data interference is carried out, i.e., by further filtering the bicluster elements determined in the previous step retaining the element values with the number of IP reads count of the sites greater than 1.5 times of the number of input reads count, which are used to carry out the estimation of the biclusters in the next step, in order to improve the model’s robustness to the low signal-to-noise ratio data, removing the interference of noise and enhancing the discriminative ability of the model. Through the above operation, elements with an IP reads count number greater than 1.5 times the number of input reads count can eventually be induced to aggregate to the corresponding bicluster, so that most of the elements finally retained in the bicluster have the characteristics of IP reads count greater than 1.5 times the number of input reads count, and all of them have an average methylation level greater than 0.6, i.e., the discovered pattern contains less unmethylation modification occurred site data, achieving the purpose of effectively removing the MeRIP-Seq sequencing noise. Its probability graphical model is shown in Fig 15. As shown in Fig 15, among the sites retained in the bicluster, only the sites that have been methylated are selected by filtering to infer the binomial distribution they follow, to further guide the data flow, thus achieving the purpose of removing sequencing noise.

The generation of data in EBBM can be viewed as first generating $\Lambda_{i}^{r}$ and $\Lambda_{j}^{c}$ from the prior distributions of the Bernoulli distribution, respectively, and then generating each site label $D_{R}=(D_{R\text{1}},D_{R\text{2}},...,D_{Rn})$ and condition label $D_{c}=(D_{C\text{1}},D_{C\text{2}},...,D_{Cm})$ from the Bernoulli distribution. Based on this label, it is determined whether it belongs to the bicluster or the background, and then the corresponding binomial distribution is selected to generate the corresponding observations, and then the pattern where the observed variables are located is adjusted according to the filtering conditions, i.e., the observed variables that satisfy the condition of $\rho^{k}>\text{0}.\text{6}$ are reprogrammed into the bicluster, and the rest are used as the background.

### 4.2. Parameter inference

As shown in Fig 15, it is difficult to estimate the parameters by maximum likelihood or maximum a posteriori methods because the model contains unobserved hidden variables. Therefore, this model uses the approximate inference Gibbs sampling method for parameter estimation. According to the Gibbs sampling method, the target variables to be sampled in this model are first determined as: 1) the hidden variable vectors of the data, $D_{R}$ and $D_{c}$, and 2) $\rho^{\text{bcl}}$and $\rho^{\text{bgd}}$. Subsequently, the full conditional probability distribution of the sampled target is determined thereby completing the inference of the parameters.

1) full-conditional probabilities of indicator variables $D_{R}$ and $D_{c}$ for site *i* and condition *j.*

Definition ${D_{R}}_{\bar{i}}=\left(D_{R\text{1}},D_{R\text{2}},...D_{R(i-\text{1})},D_{R(i+\text{1})},...D_{Rn}\right)$ denotes the vector of indicator variables for the site other than site *i*. Since ${D_{R}}_{i}$ follows the Bernoulli distribution with a probability $\Lambda_{i}^{r}$, $\Lambda_{i}^{r}$ is the probability of ${D_{R}}_{i}=\text{1}$

Therefore, the full-conditional probability distribution of $\Lambda_{i}^{r}$belonging to the bicluster for site *i* is shown in Eq. (7).


$$\begin{array}{cc} & \Lambda_{i}^{r}=p\left({D_{R}}_{i}=\text{1}|{D_{R}}_{\bar{i}},D_{C},D,\rho^{\text{bcl}},\rho^{\text{bgd}},\Lambda^{r},\Lambda^{c}\right)\;\\ & \;\;\propto p\left(D|{D_{R}}_{i}=\text{1},{D_{R}}_{\bar{i}},D_{C},\rho^{\text{bcl}},\rho^{\text{bgd}},\Lambda^{r},\Lambda^{c}\right)\cdot \\ & \;\;\;\;\;\;\;\;p\left({D_{R}}_{i}=\text{1}|\Lambda_{i}^{r}\right)\cdot p\left({D_{R}}_{\bar{i}}|\Lambda_{\bar{i}}^{r}\right)\cdot p\left(\Lambda^{r}|\zeta^{r}\right)\;\\ & \;\;=p(D|{D_{R}}_{i}=\text{1},{D_{R}}_{\bar{i}},D_{C},\rho^{\text{bcl}},\rho^{\text{bgd}},\Lambda^{r},\Lambda^{c})\cdot p({D_{R}}_{i}=\text{1},{D_{R}}_{\bar{i}}|\zeta^{r}) \end{array}$$ (7)

Where, $\Lambda^{r}=\{\Lambda_{i}^{r},\Lambda_{\bar{i}}^{r}\}$ represents the set of parameters of the Bernoulli distribution that the labels of all sites follow. $\Lambda_{\bar{i}}^{r}=\{\Lambda_{\text{1}}^{r},\Lambda_{\text{2}}^{r},...,\Lambda_{i-\text{1}}^{r},\Lambda_{i+\text{1}}^{r},...,\Lambda_{n}^{r}\}$ denotes the set of parameters of the Bernoulli distribution that the other sites follow, except for the *i*-th site. $\Lambda^{c}=\{\Lambda_{\text{1}}^{c},\Lambda_{\text{2}}^{c},...,\Lambda_{m}^{c}\}$ denotes the set of parameters of the Bernoulli distribution that the label vectors of all experimental conditions follow. In Eq. (8),


$$\begin{array}{c} p({D_{R}}_{i}=\text{1},{D_{R}}_{\bar{i}}|\zeta^{r})\propto \frac{\Gamma (v\bar{i}+\text{1}+\zeta_{\text{0}}^{r})\Gamma (n-\text{1}-v\bar{i}+\zeta_{\text{1}}^{r})}{\Gamma (\zeta_{\text{0}}^{r}+n+\overset{r}{\underset{\text{1}}{\zeta }})} \end{array}$$ (8)

$v\bar{i}$ represents the number of residual sites where the indicator variable is 1, excluding site *i*. $\overset{r}{\underset{\text{0}}{\zeta }}$ and $\overset{r}{\underset{\text{1}}{\zeta }}$ are the two shape parameters of the prior distribution.


$$\begin{array}{cc} & p\left(D|{D_{R}}_{i}=\text{1},{D_{R}}_{\bar{i}},D_{C},\rho^{\text{bcl}},\rho^{\text{bgd}},\Lambda^{r},\Lambda^{c}\right)=\;\\ & \;\;\;\;\;\;\;\prod_{\;\;\;\;\{i,j|{D_{R}}_{i}=\text{1},D_{Cj}=\text{1}\}}Binomial(a_{i,j}|n=a_{i,j}+b_{i,j},p=\rho^{\text{bcl}})\;\\ & \;\;\;\;\cdot \prod_{\left\{\overset{―}{i},j|D_{\text{R}\bar{\text{i}}}=\text{1},D_{Cj}=\text{1}\right\}}Binomial\left(a_{\overset{―}{i},j}|n=a_{\overset{―}{i},j}+b_{\overset{―}{i},j},p=\rho^{\text{bcl}}\right)\;\\ & \;\;\;\cdot \prod_{\left\{i,j|{D_{R}}_{i}=\text{1},D_{Cj}=\text{0}\right\}}Binomial\left(a_{i,j}|n=a_{i,j}+b_{i,j},p=\rho^{\text{bgd}}\right)\;\\ & \;\;\;\cdot \prod_{\left\{\overset{―}{i},j|D_{\text{R}\bar{\text{i}}}=\text{1},D_{Cj}=\text{0}\right\}}Binomial(a_{\overset{―}{i},j}|n=a_{\overset{―}{i},j}+b_{\overset{―}{i},j},p=\rho^{\text{bgd}}) \end{array}$$ (9)

Where, $i=\text{1},\text{2},...,n;\overset{―}{i}=\{\text{1},\text{2},...,(i-\text{1}),(i+\text{1}),...,n\};j=\{\text{1},\text{2},...,m\}$

Similarly, the full-conditional distribution of site *i* belonging to the background is as Eq. (10).


$$\begin{array}{c} \text{1}-\Lambda_{i}^{r}=p\left({D_{R}}_{i}=\text{0}|{D_{R}}_{\bar{i}},D_{C},D,\rho^{\text{bcl}},\rho^{\text{bgd}},\Lambda^{r},\Lambda^{c}\right)\;\propto p(D|{D_{R}}_{i}=\text{0},{D_{R}}_{\bar{i}},D_{C},\rho^{\text{bcl}},\rho^{\text{bgd}})\cdot p\left({D_{R}}_{i}=\text{0},{D_{R}}_{\bar{i}}|\zeta^{r}\right) \end{array}$$ (10)

The two factors in Eq. (10) are calculated as follows.


$$\begin{array}{c} p\left({D_{R}}_{i}=\text{0},{D_{R}}_{\bar{i}}|\zeta^{r}\right)\;\propto \frac{\Gamma (v\bar{i}+\overset{r}{\underset{\text{0}}{\zeta }})\Gamma (n-v\bar{i}+\overset{r}{\underset{\text{1}}{\zeta }})}{\Gamma (\overset{r}{\underset{\text{0}}{\zeta }}+n+\overset{r}{\underset{\text{1}}{\zeta }})} \end{array}$$ (11)

$$\begin{array}{cc} & p\left(D|{D_{R}}_{i}=\text{0},{D_{R}}_{\bar{i}},D_{C},\rho^{\text{bcl}},\rho^{\text{bgd}}\Lambda^{r},\Lambda^{c}\right)=\;\;\;\;\;\\ & \prod_{\left\{\overset{―}{i,}j|D_{\text{R}\bar{\text{i}}}=\text{1},D_{Cj}=\text{1}\right\}}Binomial(a_{\overset{―}{i,}j}\left|n=a_{\overset{―}{i},j}+b_{\overset{―}{i},j},p=\rho^{\text{bcl}}\right)\;\\ & \cdot \prod_{\left\{\overset{―}{i,}j|D_{\text{R}\bar{\text{i}}}=\text{1},D_{Cj}=\text{0}\right\}}Binomial\;\left(a_{\overset{―}{i,}j}|n=a_{\overset{―}{i,}j}+b_{\overset{―}{i},j},p=\rho^{\text{bgd}}\right)\;\;\;\;\\ & \cdot \prod_{\left\{\overset{―}{i|}D_{\text{R}\bar{\text{i}}}=\text{0}\right\}}Binomial\left(a_{\overset{―}{i,}}.|n=a_{\overset{―}{i,}}.+b_{\overset{―}{i,}}.,p=\rho^{\text{bgd}}\right)\;\;\;\;\\ & \cdot \prod_{\left\{i|{D_{R}}_{i}=\text{0}\right\}}Binomial\;(a_{i}.|n=a_{i}.+b_{i}.,p=\rho^{\text{bgd}}) \end{array}$$ (12)

Where,$i=\text{1},\text{2},...,n;\overset{―}{i}=\{\text{1},\text{2},...,(i-\text{1}),(i+\text{1}),...,n\};j=\{\text{1},\text{2},...,m\}$, $a_{i,}.$ denotes ${a_{i,}}_{j}$, where, j={1,2,...,m}, $b_{i,}.$ denotes ${b_{i,}}_{j}$, where, j={1,2,...,m}.

For convenience, we directly compute Eq. (13).


$$\begin{array}{c} log\overset{r}{\underset{i}{\gamma }}=log\frac{\Lambda_{i}^{r}}{\text{1}-\Lambda_{i}^{r}}\;=log\frac{p({D_{R}}_{i}=\text{1}|{D_{R}}_{\bar{i}},D_{C},D,\rho^{\text{bcl}},\rho^{\text{bgd}},\Lambda^{r},\Lambda^{c})}{p({D_{R}}_{i}=\text{0}|{D_{R}}_{\bar{i}},D_{C},D,\rho^{\text{bcl}},\rho^{\text{bgd}},\Lambda^{r},\Lambda^{c})}\;\propto log\frac{p(D|{D_{R}}_{i}=\text{1},{D_{R}}_{\bar{i}},D_{C},\rho^{\text{bcl}},\rho^{\text{bgd}}\Lambda^{r},\Lambda^{c})\cdot p({D_{R}}_{i}=\text{1},{D_{R}}_{\bar{i}}|\zeta^{r})}{p(D|{D_{R}}_{i}=\text{0},{D_{R}}_{\bar{i}},D_{C},\rho^{\text{bcl}},\rho^{\text{bgd}}\Lambda^{r},\Lambda^{c})\cdot p({D_{R}}_{i}=\text{0},{D_{R}}_{\bar{i}}|\zeta^{r})} \end{array}$$ (13)

Substituting Eq. (7), (8), (9), (10), (11) and (12) into Eq. (13) gives the following result,


$$\begin{array}{c} log\overset{r}{\underset{i}{\gamma }}=\sum_{\left\{i,j|D_{Ri}=\text{1},D_{Cj}=\text{1}\right\}}log\frac{Binomial(a_{i,j}|n=a_{i,j}+b_{i,j},p=\rho^{\text{bcl}})}{Binomial(a_{i,j}|n=a_{i,j}+b_{i,j},p=\rho^{\text{bgd}})}\;+log\frac{\left(v\bar{i}+\overset{r}{\underset{\text{0}}{\zeta }}\right)}{\left(n-v\bar{i}+\overset{r}{\underset{\text{1}}{\zeta }}-\text{1}\right)} \end{array}$$ (14)

From Eq. (13), we can solve for,


$$\begin{array}{c} \Lambda_{i}^{r}=\frac{e^{\overset{r}{\underset{i}{\gamma }}}}{\text{1}+e^{\overset{r}{\underset{i}{\gamma }}}} \end{array}$$ (15)

Similarly, for condition *j*, the calculation is as follows.


$$\begin{array}{c} log\overset{c}{\underset{j}{\gamma }}=log\frac{\Lambda_{j}^{c}}{\text{1}-\Lambda_{j}^{c}}\;=\sum_{\left\{i,j|D_{Ri}=\text{1},D_{Cj}=\text{1}\right\}}log\frac{Binomial(a_{i,j}|n=a_{i,j}+b_{i,j},p=\rho^{\text{bcl}})}{Binomial(a_{i,j}|n=a_{i,j}+b_{i,j},p=\rho^{\text{bgd}})}\;\;\;\;+log\frac{\left(w\bar{j}+\overset{c}{\underset{\text{0}}{\zeta }}\right)}{\left(m-w\bar{j}+\overset{c}{\underset{\text{1}}{\zeta }}-\text{1}\right)} \end{array}$$ (16)

$w\bar{j}$ represents the number of remaining conditions where the indicator variable is 1, except for condition *j*. $\overset{c}{\underset{\text{0}}{\zeta }}$ and $\overset{c}{\underset{\text{1}}{\zeta }}$ are the two shape parameters of the prior distribution of $\Lambda_{j}^{c}$.

From Equation (16), we can solve for,


$$\begin{array}{c} \Lambda_{j}^{c}=\frac{e^{\overset{c}{\underset{j}{\gamma }}}}{\text{1}+e^{\overset{c}{\underset{j}{\gamma }}}} \end{array}$$ (17)

2) Full-conditional probability distribution of $\rho^{\text{bcl}}$and $\rho^{\text{bgd}}$of bicluster and background based on data screening strategy.

First, calculate the full-conditional probability distribution of $\rho^{\text{bcl}}$ without data filtering, and then perform data filtering. The full-conditional probability of $\rho^{\text{bcl}}$ is derived as follows using Bayes’ formula.


$$\begin{array}{cc} & p\left(p=\rho^{\text{bcl}}|D,D_{R},D_{C},\rho^{\text{bgd}},\zeta^{r},\zeta^{c}\right)\;=\frac{p(D|p=\rho^{\text{bcl}},D_{R},D_{C},\rho^{\text{bgd}},\zeta^{r},\zeta^{c})\cdot p(\rho^{\text{bcl}})}{\int p(D|p=\rho^{\text{bcl}},D_{R},D_{C},\rho^{\text{bgd}},\zeta^{r},\zeta^{c})\cdot p(\rho^{\text{bcl}})d\rho^{\text{bcl}}}\;\\ & \;\;=\frac{\prod_{\left\{i,j|i\in I,j\in J\right\}}\left(\rho^{\text{bcl}}{)}^{a_{i,j}}\cdot (\text{1}-\rho^{\text{bcl}}{)}^{b_{i,j}}\cdot (\rho^{\text{bcl}}{)}^{\alpha^{\text{bcl}}-\text{1}}\cdot (\text{1}-\rho^{\text{bcl}}{)}^{\beta^{\text{bcl}}-\text{1}}\cdot Binomial\;({𝐃}_{\text{bgd}}|\rho^{\text{bgd}}\right)}{Binomial({𝐃}_{\text{bgd}}|\rho^{\text{bgd}})\cdot \int \prod_{\left\{i,j|i\in I,j\in J\right\}}(\rho^{\text{bcl}}{)}^{a_{i,j}}\cdot (\text{1}-\rho^{\text{bcl}}{)}^{b_{i,j}}\cdot (\rho^{\text{bcl}}{)}^{\alpha^{\text{bcl}}-\text{1}}\cdot (\text{1}-\rho^{\text{bcl}}{)}^{\beta^{\text{bcl}}-\text{1}}d\rho^{\text{bcl}}}\;\\ & \;\;=\frac{(\rho^{\text{bcl}}{)}^{\left(\alpha^{\text{bcl}}-\text{1}+\sum_{\left\{i,j|i\in I,j\in J\right\}}a_{i,j}\right)}\cdot (\text{1}-\rho^{\text{bcl}}{)}^{\left(\beta^{\text{bcl}}-\text{1}+\sum_{\left\{i,j|i\in I,j\in J\right\}}b_{i,j}\right)}}{\int (\rho^{\text{bcl}}{)}^{\left(\alpha^{\text{bcl}}-\text{1}+\sum_{\left\{i,j|i\in I,j\in J\right\}}a_{i,j}\right)}\cdot (\text{1}-\rho^{\text{bcl}}{)}^{\left(\beta^{\text{bcl}}-\text{1}+\sum_{\left\{i,j|i\in I,j\in J\right\}}b_{i,j}\right)}d\rho^{\text{bcl}}\;}\;\\ & \;\;=\frac{\frac{\Gamma \left(\alpha^{\text{bcl}}+\sum_{\left\{i,j|i\in I,j\in J\right\}}a_{i,j}\right)\Gamma \left(\beta^{\text{bcl}}+\sum_{\left\{i,j|i\in I,j\in J\right\}}b_{i,j}\right)}{\Gamma \left(\alpha^{\text{bcl}}+\sum_{\left\{i,j|i\in I,j\in J\right\}}a_{i,j}+\beta^{\text{bcl}}+\sum_{\left\{i,j|i\in I,j\in J\right\}}b_{i,j}\right)}\cdot \text{Beta}\left(\rho^{\text{bcl}}|\alpha^{\text{bcl}}+\sum_{\left\{i,j|i\in I,j\in J\right\}}a_{i,j},\beta^{\text{bcl}}+\sum_{\left\{i,j|i\in I,j\in J\right\}}b_{i,j}\right)}{\frac{\Gamma \left(\alpha^{\text{bcl}}+\sum_{\left\{i,j|i\in I,j\in J\right\}}a_{i,j}\right)\Gamma \left(\beta^{\text{bcl}}+\sum_{\left\{i,j|i\in I,j\in J\right\}}b_{i,j}\right)}{\Gamma \left(\alpha^{\text{bcl}}+\sum_{\left\{i,j|i\in I,j\in J\right\}}a_{i,j}+\beta^{\text{bcl}}+\sum_{\left\{i,j|i\in I,j\in J\right\}}b_{i,j}\right)}}\;\\ & \;\;=\text{Beta}\left(\rho^{\text{bcl}}|\alpha^{\text{bcl}}+\sum_{\left\{i,j|i\in I,j\in J\right\}}a_{i,j},\;\;\;\;\beta^{\text{bcl}}+\sum_{\left\{i,j|i\in I,j\in J\right\}}b_{i,j}\right) \end{array}$$ (18)

I⊂{1,...,n},J⊂{1,...,m} indicate the set of sites and conditions included in the bicluster.

According to the screening strategy, the next step is to screen out the set of sites and conditions that satisfy the condition that the number of IP sample reads count at the corresponding experimental conditions is greater than 1.5 times the corresponding input reads count, i.e., *I’* and *J*’. *I’* and *J’* are subsets of *I* and *J*, respectively. The elements contained in *I’* and *J’* all satisfy $a_{i,j}>\text{1}.\text{5}\times b_{i,j}$, that is, their methylation levels are all greater than 0.6. The full-conditions probability distribution of $\rho^{\text{bcl}}$after screening is shown in Eq. (19).


$$\begin{array}{c} p\left(p=\rho^{\text{bcl}}|D,D_{R},D_{C},\rho^{\text{bgd}},\zeta^{r},\zeta^{c}\right)\;=\text{Beta}\left(\rho^{\text{bcl}}|\alpha^{\text{bcl}}+\sum_{\left\{i,j|i\in I^{\prime },j\in J^{\prime }\right\}}a_{i,j},\;\;\;\;\beta^{\text{bcl}}+\sum_{\left\{i,j|i\in I^{\prime },j\in J^{\prime }\right\}}b_{i,j}\right) \end{array}$$ (19)

Similarly, the full-conditional probability of unscreened $\rho^{\text{bgd}}$ is derived as follows.


$$\begin{array}{cc} & p\left(p=\rho^{\text{bgd}}|D,D_{R},D_{C},\rho^{\text{bcl}},\zeta^{r},\zeta^{c}\right)\\ & \;\;\;=\frac{p(D|p=\rho^{\text{bgd}},D_{R},D_{C},\rho^{\text{bcl}},\zeta^{r},\zeta^{c})\cdot p(\rho^{\text{bgd}})}{\int p(D|p=\rho^{\text{bgd}},D_{R},D_{C},\rho^{\text{bcl}},\zeta^{r},\zeta^{c})\cdot p(\rho^{\text{bgd}})d\rho^{\text{bgd}}}\;\\ & \;\;\;=\frac{\prod_{\left\{i,j|i\notin I,j\notin J\right\}}\left(\rho^{\text{bgd}}{)}^{a_{i,j}}\cdot (\text{1}-\rho^{\text{bgd}}{)}^{b_{i,j}}\cdot (\rho^{\text{bgd}}{)}^{\left(\alpha^{\text{bgd}}-\text{1}\right)}\cdot (\text{1}-\rho^{\text{bgd}}{)}^{\left(\beta^{\text{bgd}}-\text{1}\right)}\cdot Binomial({𝐃}_{\text{bcl}}|\rho^{\text{bcl}}\right)}{Binomial({𝐃}_{\text{bcl}}|\rho^{\text{bcl}})\int \prod_{\left\{i,j|i\notin I,j\notin J\right\}}(\rho^{\text{bgd}}{)}^{a_{i,j}}\cdot (\text{1}-\rho^{\text{bgd}}{)}^{b_{i,j}}\cdot (\rho^{\text{bgd}}{)}^{\left(\alpha^{\text{bgd}}-\text{1}\right)}\cdot (\text{1}-\rho^{\text{bgd}}{)}^{\left(\beta^{\text{bgd}}-\text{1}\right)}d\rho^{\text{bgd}}}\;\\ & \;\;\;=\frac{(\rho^{\text{bgd}}{)}^{\left(\alpha^{\text{bgd}}-\text{1}+\sum_{\left\{i,j|i\in I,j\in J\right\}}a_{i,j}\right)}\cdot (\text{1}-\rho^{\text{bgd}}{)}^{\left(\beta^{\text{bgd}}-\text{1}+\sum_{\left\{i,j|i\in I,j\in J\right\}}b_{i,j}\right)}}{(\rho^{\text{bgd}}{)}^{\left(\alpha^{\text{bgd}}-\text{1}+\sum_{\left\{i,j|i\notin I,j\notin J\right\}}a_{i,j}\right)}\cdot (\text{1}-\rho^{\text{bgd}}{)}^{\left(\beta^{\text{bgd}}-\text{1}+\sum_{\left\{i,j|i\notin I,j\notin J\right\}}b_{i,j}\right)}d\rho^{\text{bgd}}}\;\\ & \;\;\;=\frac{\frac{\Gamma \left(\alpha^{\text{bgd}}+\sum_{\left\{i,j|i\notin I,j\notin J\right\}}a_{i,j}\right)\Gamma \left(\beta^{\text{bgd}}+\sum_{\left\{i,j|i\notin I,j\notin J\right\}}b_{i,j}\right)}{\Gamma \left(\alpha^{\text{bgd}}+\sum_{\left\{i,j|i\notin I,j\notin J\right\}}a_{i,j},+\beta^{\text{bgd}}+\sum_{\left\{i,j|i\notin I,j\notin J\right\}}b_{i,j}\right)}\text{Beta}\left(\rho^{\text{bgd}}|\alpha^{\text{bgd}}+\sum_{\left\{i,j|i\notin I,j\notin J\right\}}a_{i,j},\beta^{\text{bgd}}+\sum_{\left\{i,j|i\notin I,j\notin J\right\}}b_{i,j}\right)}{\frac{\Gamma \left(\alpha^{\text{bgd}}+\sum_{\left\{i,j|i\notin I,j\notin J\right\}}a_{i,j}\right)\Gamma \left(\beta^{\text{bgd}}+\sum_{\left\{i,j|i\notin I,j\notin J\right\}}b_{i,j}\right)}{\Gamma \left(\alpha^{\text{bgd}}+\sum_{\left\{i,j|i\notin I,j\notin J\right\}}a_{i,j},+\beta^{\text{bgd}}+\sum_{\left\{i,j|i\notin I,j\notin J\right\}}b_{i,j}\right)}}\;\\ & =\text{Beta}\left(\rho^{\text{bgd}}|\alpha^{\text{bgd}}+\sum_{\left\{i,j|i\notin I,j\notin J\right\}}a_{i,j},\;\;\;\;\beta^{\text{bgd}}+\sum_{\left\{i,j|i\notin I,j\notin J\right\}}b_{i,j}\right) \end{array}$$ (20)

{i,j|i∉I,j∉J} represents the set of sites and conditions included in the background.

The full-conditions probability distribution of $\rho^{\text{bgd}}$ after screening is shown in Eq. (21).


$$\begin{array}{c} p\left(p=\rho^{\text{bgd}}|D,D_{R},D_{C},\rho^{\text{bcl}},\zeta^{r},\zeta^{c}\right)\;=\text{Beta}\left(\rho^{\text{bgd}}|\alpha^{\text{bgd}}+\sum_{\left\{i,j|i\notin I^{\prime },j\notin J^{\prime }\right\}}a_{i,j},\;\;\;\;\beta^{\text{bgd}}+\sum_{\left\{i,j|i\notin I^{\prime },j\notin J^{\prime }\right\}}b_{i,j}\right) \end{array}$$ (21)

### 4.3. Algorithm implementation

1) Implementation of a probabilistic model for a single bicluster

The implementation of the probability model for a single bicluster consists of three parts: the design of the Gibbs sampler, convergence judgment, and final pattern determination. The specific implementation is as follows.

(1) Design of Gibbs sampler

**Fig 16:**
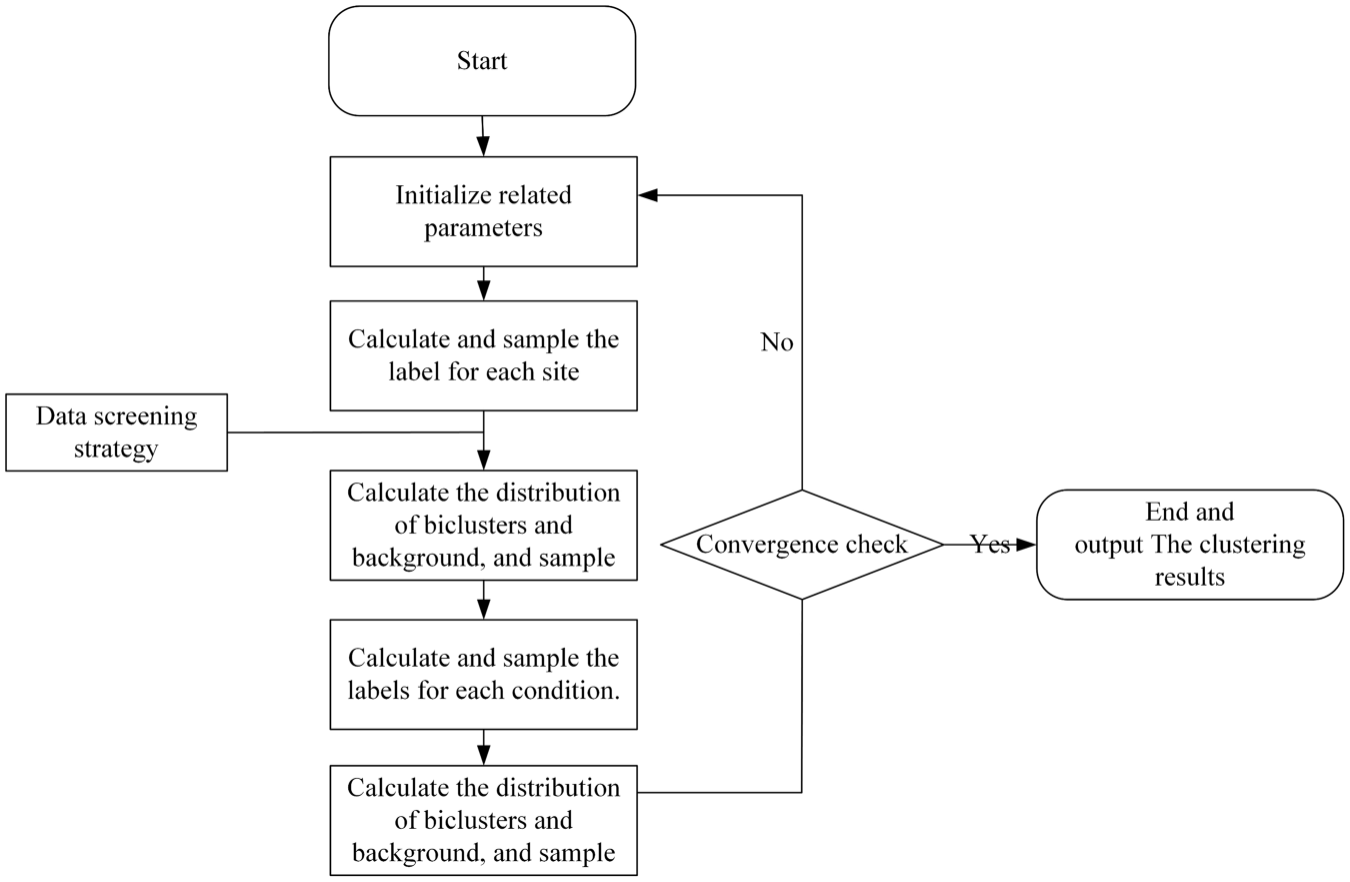
Algorithmic framework for a single bicluster probability model based on data screening strategies.

Based on the above, the algorithmic framework for a single bicluster probability model can be designed as Fig 16.

Compared with the BBM model, the EBBM model adds a data screening strategy in step 2 for noise reduction. First, based on **D**_**R**_ and **D**_**C**_, screen out the set of sites and conditions belonging to the bicluster, i.e., *I* and *J*. Then, further screen the selected sites and conditions to select the set of sites and conditions that satisfy the condition that the number of IP sample reads count at the corresponding experimental conditions is greater than 1.5 times the corresponding input reads count, i.e., *I’* and *J’*. Finally, substitute into Eq. (19) to calculate the binomial distribution of the bicluster. Comparing Eq. (19) and Eq. (18), we can see that the incremental terms of the two shape parameters of the beta distribution in Eq. (19) are subsets of the two shape parameters of the beta distribution in Eq. (18). The expectation value of the beta distribution represented by Eq. (19) can be expressed as Eq. (22).


$$\begin{array}{c} E(\rho^{\text{bcl}})=\frac{\alpha^{\text{bcl}}+\sum_{\left\{i,j|i\in I^{\prime },j\in J^{\prime }\right\}}a_{i,j}}{\alpha^{\text{bcl}}+\sum_{\left\{i,j|i\in I^{\prime },j\in J^{\prime }\right\}}a_{i,j}+\beta^{\text{bcl}}+\sum_{\left\{i,j|i\in I^{\prime },j\in J^{\prime }\right\}}b_{i,j}} \end{array}$$ (22)

In Eq. (22), $\alpha^{\text{bc1}}$ and $\beta^{\text{bc1}}$ are hyperparameters, and their values can be ignored compared to the values of $\sum_{\left\{i,j|i\in I^{\prime },j\in J^{\prime }\right\}}a_{i,j}$ and $\sum_{\left\{i,j|i\in I^{\prime },j\in J^{\prime }\right\}}b_{i,j}$. Therefore, the value of Eq. (22) can be approximated by Eq. (23).


$$\begin{array}{c} E(\rho^{\text{bcl}})\approx \frac{\sum_{\left\{i,j|i\in I^{\prime },j\in J^{\prime }\right\}}a_{i,j}}{\sum_{\left\{i,j|i\in I^{\prime },j\in J^{\prime }\right\}}a_{i,j}+\sum_{\left\{i,j|i\in I^{\prime },j\in J^{\prime }\right\}}b_{i,j}} \end{array}$$ (23)

In Eq. (23), since the retained elements are all elements where $a_{i,j}>\text{1}.\text{5}\times b_{i,j}$, the expectation estimated by this formula is at least greater than 0.6, its value is larger than that of formula (18). In other words, the sites retained after screening the temporarily obtained biclusters in the iterative process using Eq. (19) are, according to the MeRIP-Seq sequencing principle, sites that have theoretically undergone methylation.

From the above, in the subsequent calculation steps, Eq. (19) needs to be substituted for Eq. (18) to calculate the binomial distribution parameters $\rho^{\text{bc1}}$ of the estimated bicluster.

Next, we estimate the conditional label. Whsen the value of $\overset{c}{\underset{j}{\Lambda }}$ is larger, the probability that condition *j* belongs to bicluster is greater. According to Eq. (17),


$$\begin{array}{c} \Lambda_{j}^{c}=\frac{e^{\overset{c}{\underset{j}{\gamma }}}}{\text{1}+e^{\overset{c}{\underset{j}{\gamma }}}}=\frac{\text{1}}{e^{-\overset{c}{\underset{j}{\gamma }}}+\text{1}} \end{array}$$ (24)

In Eq. (24), since $f(x)=\;e^{-x}$ is a monotonically decreasing function, the larger the value of $\overset{c}{\underset{j}{\gamma }}$, the smaller the value of $e^{-\overset{c}{\underset{j}{\gamma }}}$, thereby causing the value of Eq. (24) to increase.

From the above, we can conclude that the larger the value of $\overset{c}{\underset{j}{\gamma }}$, the larger the value of $\overset{c}{\underset{j}{\Lambda }}$ will be, and $\overset{c}{\underset{j}{\Lambda }}$and $\overset{c}{\underset{j}{\gamma }}$ are directly proportional to each other.

Again, by Eq. (16)


$$\begin{array}{c} log\overset{c}{\underset{j}{\gamma }}=\;\sum_{\left\{i,j|D_{Ri}=\text{1},D_{Cj}=\text{1}\right\}}log\frac{Binomial\;(a_{i,j}|n=a_{i,j}+b_{i,j},p=\rho^{\text{bcl}})}{Binomial\;(a_{i,j}|n=a_{i,j}+b_{i,j},p=\rho^{\text{bgd}})}\;\;\;\;+log\frac{\left(w\bar{j}+\overset{c}{\underset{\text{0}}{\zeta }}\right)}{\left(m-w\bar{j}+\overset{c}{\underset{\text{1}}{\zeta }}-\text{1}\right)} \end{array}$$ (25)

In step 3 of the Gibbs sampler, for the first experimental condition at the start, since **D**_**R**_ and **D**_**Cj**_ are fixed in advance, the value $log\frac{\left(w\bar{j}+\overset{c}{\underset{\text{0}}{\zeta }}\right)}{\left(m-w\bar{j}+\overset{c}{\underset{\text{1}}{\zeta }}-\text{1}\right)}$ in Eq. (25) does not change with the value of $\rho^{\text{bc1}}$. Therefore, the value of formula (25) isdetermined by $\sum_{\left\{i,j|D_{Ri}=\text{1},D_{Cj}=\text{1}\right\}}log\frac{Binomial\;(a_{i,j}|n=a_{i,j}+b_{i,j},p=\rho^{\text{bcl}})}{Binomial\;(a_{i,j}|n=a_{i,j}+b_{i,j},p=\rho^{\text{bgd}})}$. After the previous data screening, the value of $\rho^{\text{bc1}}$ isrelatively large. When the value of $\rho^{\text{bc1}}$ is large, the probability density function of the binomial distribution shows that when the value of $a_{i,j}$ is large, the value of $\text{Binomial}\;(a_{i,j}|n=a_{i,j}+b_{i,j},p=\rho^{\text{bcl}})$ is large, while the value of $\text{Binomial}\;(a_{i,j}|n=a_{i,j}+b_{i,j},p=\rho^{\text{bgd}})$ is small. In other words, $a_{i,j}$ tends to come from the binomial distribution with larger$\rho^{\text{bc1}}$ value. Then the value of $\frac{Binomial\;(a_{i,j}|n=a_{i,j}+b_{i,j},p=\rho^{\text{bcl}})}{Binomial\;(a_{i,j}|n=a_{i,j}+b_{i,j},p=\rho^{\text{bgd}})}$ will be larger. Conversely, the smaller $a_{i,j}$ is, the smaller the value of $\frac{Binomial\;(a_{i,j}|n=a_{i,j}+b_{i,j},p=\rho^{\text{bcl}})}{Binomial\;(a_{i,j}|n=a_{i,j}+b_{i,j},p=\rho^{\text{bgd}})}$will be. Since the log function is a monotonically increasing function, in this step, when $\rho^{\text{bc1}}$ is large, Eq. (25) will estimate the label for the first experimental condition based on the relationship between $a_{i,j}$ and $a_{i,j}+b_{i,j}$ in the already determined bicluster. If most of the data satisfy $a_{i,j}>\text{1}.\text{5}\times b_{i,j}$, then Eq. (25) will give $\overset{c}{\underset{j}{\gamma }},j=\text{1}$ a larger value. From the results derived above, $\Lambda_{j}^{c}$ is directly proportional to $\overset{c}{\underset{j}{\gamma }}$, so the larger the value of $\overset{c}{\underset{j}{\gamma }},j=\text{1}$, the greater the probability of sampling the corresponding label as 1, meaning that it is more likely to belong to the bicluster.

Then, for the next $\overset{c}{\underset{j}{\gamma }},j=\text{2}$, $log\frac{\left(w\bar{j}+\overset{c}{\underset{\text{0}}{\zeta }}\right)}{\left(m-w\bar{j}+\overset{c}{\underset{\text{1}}{\zeta }}-\text{1}\right)}$ will increase, and the value of $\sum_{\left\{i,j|D_{Ri}=\text{1},D_{Cj}=\text{1}\right\}}log\frac{Binomial\;(a_{i,j}|n=a_{i,j}+b_{i,j},p=\rho^{\text{bcl}})}{Binomial\;(a_{i,j}|n=a_{i,j}+b_{i,j},p=\rho^{\text{bgd}})}$ will be determined according to whether the elements added to the bicluster based on the first experimental condition added still satisfy the condition that $a_{i,j}>\text{1}.\text{5}\times b_{i,j}$ in most cases. If most elements still have the property $a_{i,j}>\text{1}.\text{5}\times b_{i,j}$, assign $\overset{c}{\underset{j}{\gamma }},j=\text{2}$ a larger value; otherwise, assign it a smaller value. Therefore, when the first experimental condition has been determined to be a bicluster, Eq. (25) will attempt to increase the number of elements to expand the size of the bicluster, and then determine whether the expanded bicluster still retains the property that most elements satisfy $a_{i,j}>\text{1}.\text{5}\times b_{i,j}$. If so, continue attempting to expand the size of the bicluster; otherwise, reduce the size of the bicluster. The determination of subsequent experimental condition labels follows the same principle.

Conversely, if, when initially evaluating the first experimental condition, most of the elements in the bicluster that has been identified do not exhibit the property $a_{i,j}>\text{1}.\text{5}\times b_{i,j}$, then $\Lambda_{j}^{c},j=\text{1}$ will be assigned a smaller value. The likelihood of the first experimental condition being assigned to the bicluster is low. In other words, the algorithm determines that the first experimental condition does not belong to the bicluster, thereby removing some elements and reducing the size of the bicluster. This allows the algorithm to statistically analyze whether most elements in the reduced-size bicluster exhibit the $a_{i,j}>\text{1}.\text{5}\times b_{i,j}$ property when evaluating the second experimental condition. If the property is present, the second experimental condition is judged to belong to the bicluster, and thus, some elements under the second experimental condition are added to the bicluster, thereby increasing the size of the bicluster when judging the third experimental condition. If the property is not satisfied, the size of the bicluster is further reduced, and the properties of the contained elements are counted to judge the third experimental condition. Subsequent experimental condition judgments follow the same principle.

The same applies to the labeling of sites. Therefore, the data screening strategy will induce elements that satisfy the filtering strategy to cluster together.

In summary, by improving the Gibbs sampler, we can eliminate the noise problem introduced by sequencing errors to a certain extent.

The Gibbs sampler for the single bicluster probability model is designed as Algorithm 1.

**Algorithm 1: Gibbs sampler for a single bicluster probability model**

**Input:** randomly initialize the label vectors **D**_R_ and **D**_**C**_ for the sites and sample conditions, the preset number of iterations *K* and the burn-in number *L*, the IP sample reads count matrix ${\left(a_{i,j}\right)}_{n\times m}$, the input sample reads count matrix ${\left(b_{i,j}\right)}_{n\times m}$, and the hyperparameters ($\overset{r}{\underset{\text{0}}{\zeta }},\overset{r}{\underset{\text{1}}{\zeta }}$), ($\overset{c}{\underset{\text{0}}{\zeta }},\overset{c}{\underset{\text{1}}{\zeta }}$), ($\alpha^{\text{bcl}},\;\beta^{\text{bcl}}$) and ($\alpha^{\text{bgd}},\;\beta^{\text{bgd}}$).

**Output:** determined biclusters and background

1.  For each site i,i=1,2,…n, fix **D**_**C**_, $\rho^{\text{bc1}}$, $\rho^{\text{bgd}}$, and the labels **D**_RT_ for other sites.

     1) Calculate the Bernoulli distribution of the site label according to (14) and (15).

     2) Sample the label *D*_*Rj*_ for the site based on the distribution.

**2.**  Fix **D**_R_ and **D**_C_

     1) Based on **D**_R_ and **D**_C_, filter out the sites and conditions belonging to the bicluster, i.e., $\left\{i|{𝐃}_{R}[i]=\text{1}\right\}=I$ and $\left\{j|{𝐃}_{C}[j]=\text{1}\right\}=J$.

     2) Further screen the selected sites *I* and conditions *J* to identify sites *I’* and conditions *J’* that satisfy $a_{i,j}>\text{1}.\text{5}\times b_{i,j}$.

     3) Calculate the binomial distribution of the bicluster and the background according to (19) and (21).

     4) Sample $\rho^{\text{bc1}}$ and $\rho^{\text{bgd}}$ according to their distribution.

**3.**  For each sample condition j,j=1,2…m, fix **D**_R_, $\rho^{\text{bc1}}$, $\rho^{\text{bgd}}$, and the labels **D**_Cj_ of other sample conditions.

     1) Calculate the Bernoulli distribution of the sample condition label according to (16) and (17).

     2) Sample the label$D_{Cj}$ for the sample conditions based on this distribution.

**4.**  Fix **D**_R_ and **D**_C_

     1) Calculate the binomial distribution of the bicluster and the background according to (18) and (20).

     2) Sample $\rho^{\text{bc1}}$ and $\rho^{\text{bgd}}$ according to their distribution.

**5.**Repeat steps 1, 2, 3, and 4 until the iteration ends.

(2) Convergence diagnosis

There are many methods for diagnosing convergence, and in practice, convergence is usually assessed from multiple perspectives. This paper references the convergence diagnosis method proposed by Liu et al [58], which involves checking the intra-chain variance after Algorithm 1 has completed the predefined number of iterations. If the convergence condition is met, the algorithm stops. Otherwise, the iteration count increases until convergence is achieved. Once convergence has been achieved, the historical trace plots of the parameters and the log-likelihood are examined to confirm the convergence of the algorithm. Eq. (26) show the method for calculating the log-likelihood function in the model.


$$\begin{array}{cc} & p\left(D|D_{R},D_{C},\rho^{\text{bcl}},\rho^{\text{bgd}}\right)\;\\ & =log\left(\begin{array}{cc} & \prod_{\left\{i,j|D_{\text{Ri}}=\text{1},D_{Cj}=\text{1}\right\}}Binomial\;(a_{i,j}|n=a_{i,j}+b_{i,j},p=\rho^{\text{bcl}})\cdot \\ & \prod_{\left\{i,j|D_{\text{Ri}}=\text{1},D_{Cj}=\text{0}\right\}}Binomial\;(a_{i,j}|n=a_{i,j}+b_{i,j},p=\rho^{\text{bgd}})\cdot \\ & \prod_{\left\{i|D_{\text{Ri}}=\text{0}\right\}}Binomial\;(a_{i,}|n=a_{i,}+b_{i,},p=\rho^{\text{bgd}}) \end{array}\right)\;\\ & =\sum_{\left\{i,j|D_{\text{Ri}}=\text{1},D_{Cj}=\text{1}\right\}}\text{log}(Binomial\;(a_{i,j}|n=a_{i,j}+b_{i,j},p=\rho^{\text{bcl}}))+\\ & \;\;\;\;\;\sum_{\left\{i,j|D_{\text{Ri}}=\text{1},D_{Cj}=\text{0}\right\}}\text{log}(Binomial\;(a_{i,j}|n=a_{i,j}+b_{i,j},p=\rho^{\text{bgd}}))\\ & +\;\;\;\;\;\sum_{\left\{i,.|D_{\text{Ri}}=\text{0}\right\}}\text{log}\;(Binomial\;(a_{i,}|n=a_{i,}+b_{i,},p=\rho^{\text{bgd}})) \end{array}$$ (26)

(3) Determination of the final pattern

To determine the final pattern, after the algorithm converges, the elements of the bicluster are determined using the Monte Carlo integration method. Specifically, the sites with site label values higher than the three-quarter quartile of all site labels are selected as the sites of the bicluster. Similarly, the conditions with condition label values higher than the three-quarter quartile of all condition labels are selected as the conditions of the bicluster. The remaining sites and conditions are treated as background elements.

2) Probabilistic model of multiple biclusters

This model uses a site non-overlap method to discover multiple biclusters. This involves masking the sites of the discovered biclusters and repeatedly executing the sampling programme on the remaining data until the algorithm terminates and determines the number of biclusters discovered. Liu et al. used this method to determine the number of biclusters. The implementation process is shown in Algorithm 2.

**Algorithm 2**

**Input:** Output results of Algorithm 1

**Output:** Rows belonging to the discovered bicluster and “yes” or “no”.

**1.**  Mask rows that have been assigned to the discovered bicluster.

**2.**  Run Algorithm 1

**3.**  Determine whether there are any remaining rows or conditions to be assigned to the new bicluster.

3) Implementation of the entire EBBM algorithm.

In summary, the entire EBBM algorithm design is shown in algorithm 3.

**Algorithm 3**

**Input:** IP sample reads count matrix $(a_{i,j}{)}_{n\times m}$, input sample reads count matrix $(b_{i,j}{)}_{n\times m}$, maximum number of biclusters *N*, and other relevant parameter initial values required by the algorithm.

**Output:** All biclusters found: *Bic*_*1*_*, Bic*_*2*_*, … Bic*_*k*_*.*

***1***. For *k* in 1: *N*

**2.**  Feed data matrices $(a_{i,j}{)}_{n\times m}$ and $(b_{i,j}{)}_{n\times m}$ into

Algorithm 1 to obtain ${BiC}_{k}$.

**3.**  Feed the output results of Algorithm 1 into

Algorithm 2.

**4.**  If: The output result of Algorithm 2 contains “yes”:

**5.**   *k = k* + 1

**6.**  else: break

## Supporting information

S1 Fig Sensitivity analysis of IP/Input ratio threshold. The graph evaluates the impact of varying the IP/Input ratio threshold on retained sites and the stability of the results. The “Retained sites” column shows the number of sites retained at each threshold. The “Stable range (1.2-2.0)” highlights the threshold interval where the core co-methylation patterns (i.e., biclusters with high stability) remained largely unchanged. The “Jaccard similarity (vs ratio=1.5)” column quantifies the overlap between the site sets obtained at each threshold and the reference set obtained at ratio = 1.5. A Jaccard similarity value above 0.8 indicates high reproducibility. Importantly, the top enriched biological processes (e.g., “Histone modification”, “peptidyl-lysine modification”) were consistently reproduced across thresholds from 1.2 to 2.0. When the ratio was set to 1.0 (i.e., no strict filtering) or 2.5 (overly stringent), the biclustering results became unstable or lost meaningful patterns. The above indicates that the 1.5 threshold is a reasonable default choice, and our algorithm’s performance is robust to moderate variations (±0.3) around this value. The analysis suggests that thresholds between 1.2 and 2.0 yield stable and consistent results. Abbreviations: IP, immunoprecipitation.
(TIF)
